# Exploring functional connectivity in clinical and data-driven groups of preterm and term adults

**DOI:** 10.1093/braincomms/fcaf074

**Published:** 2025-02-17

**Authors:** Laila Hadaya, František Váša, Konstantina Dimitrakopoulou, Mansoor Saqi, Sukhwinder S Shergill, A David Edwards, Dafnis Batalle, Robert Leech, Chiara Nosarti

**Affiliations:** Department of Early Life Imaging, School of Biomedical Engineering & Imaging Sciences, King’s College London, London SE1 7EH, UK; Department of Child and Adolescent Psychiatry, Institute of Psychiatry Psychology and Neuroscience (IoPPN), King’s College London, London SE5 8AB, UK; Department of Neuroimaging, IoPPN, King’s College London, London SE5 8AB, UK; Translational Bioinformatics Platform, NIHR Biomedical Research Centre, Guy’s and St. Thomas’ NHS Foundation Trust and King’s College London, London SE1 9RT, UK; Translational Bioinformatics Platform, NIHR Biomedical Research Centre, Guy’s and St. Thomas’ NHS Foundation Trust and King’s College London, London SE1 9RT, UK; Kent and Medway Medical School, Canterbury CT2 7FS, UK; Kent and Medway NHS and Social Care Partnership Trust, Maidstone ME16 9PH, UK; Department of Psychosis Studies, IoPPN, King’s College London, London SE5 8AB, UK; Department of Early Life Imaging, School of Biomedical Engineering & Imaging Sciences, King’s College London, London SE1 7EH, UK; Department of Early Life Imaging, School of Biomedical Engineering & Imaging Sciences, King’s College London, London SE1 7EH, UK; Department of Forensic and Neurodevelopmental Sciences, IoPPN, King’s College London, London SE5 8AB, UK; Department of Neuroimaging, IoPPN, King’s College London, London SE5 8AB, UK; Department of Early Life Imaging, School of Biomedical Engineering & Imaging Sciences, King’s College London, London SE1 7EH, UK; Department of Child and Adolescent Psychiatry, Institute of Psychiatry Psychology and Neuroscience (IoPPN), King’s College London, London SE5 8AB, UK

**Keywords:** preterm birth, clustering, resting state functional MRI, behaviour

## Abstract

Adults born very preterm (i.e. at <33 weeks’ gestation) are more susceptible to long-lasting structural and functional brain alterations and cognitive and socio-emotional difficulties, compared with full-term controls. However, behavioural heterogeneity within very preterm and full-term individuals makes it challenging to find biomarkers of specific outcomes. To address these questions, we parsed brain–behaviour heterogeneity in participants subdivided according to their clinical birth status (very preterm versus full term) and/or data-driven behavioural phenotype (regardless of birth status). Participants were followed-up in adulthood (median age 30 years) as part of a wider longitudinal case–control cohort study. The Network Based Statistic approach was used to identify topological components of resting state functional connectivity differentiating between (i) 116 very preterm and 83 full-term adults (43% and 57% female, respectively) and (ii) data-driven behavioural subgroups identified using consensus clustering (*n* = 156, 46% female). Age, sex, socio-economic status and in-scanner head motion were used as confounders in all analyses. *Post hoc* two-way group interactions between clinical birth status and behavioural data-driven subgrouping classification labels explored whether functional connectivity differences between very preterm and full-term adults varied according to distinct behavioural outcomes. Very preterm compared with full-term adults had poorer scores in selective measures of cognitive and socio-emotional processing and displayed complex patterns of hyper- and hypo-connectivity in sub-sections of the default mode, visual and ventral attention networks. Stratifying the study participants in terms of their behavioural profiles (irrespective of birth status) identified two data-driven subgroups: an ‘At-Risk’ subgroup, characterized by increased cognitive, mental health and socio-emotional difficulties, displaying hypo-connectivity anchored in frontal opercular and insular regions, relative to a ‘Resilient’ subgroup with more favourable outcomes. No significant interaction was noted between clinical birth status and behavioural data-driven subgrouping classification labels in terms of functional connectivity. Functional connectivity differentiating between very preterm and full-term adults was dissimilar to functional connectivity differentiating between the data-driven behavioural subgroups. We speculate that functional connectivity alterations observed in very preterm relative to full-term adults may confer both risk and resilience to developing behavioural sequelae associated with very preterm birth, while the localized functional connectivity alterations seen in the ‘At-Risk’ subgroup relative to the ‘Resilient’ subgroup may underlie less favourable behavioural outcomes in adulthood, irrespective of birth status.

## Teaser

Hadaya *et al*. parsed brain–behaviour heterogeneity in adults subdivided according to their clinical birth status (very preterm versus full term) and/or data-driven behavioural phenotype (regardless of birth status). They identified alterations in resting state functional connectivity associated with very preterm birth that differed from those underlying less favourable behavioural outcomes.

## Graphical Abstract

**Graphical Abstract:**
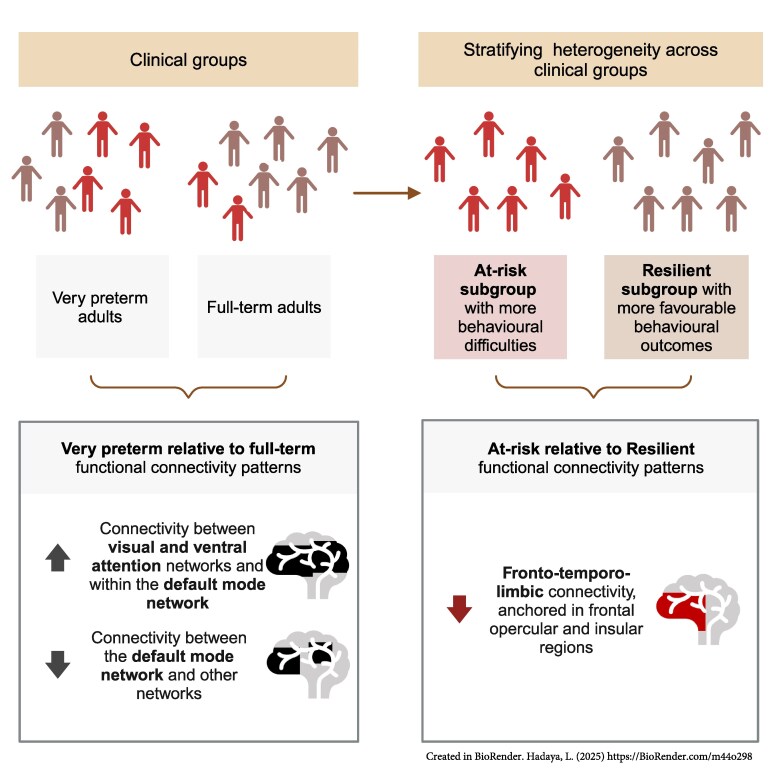

## Introduction

Very preterm (VPT) birth (i.e. at <33 weeks’ gestation) occurs during a rapid stage of brain development, making those born VPT vulnerable to neurological insult^1^ and long-lasting difficulties in attention, executive function and socio-emotional processing.^2-4^ Functional connectivity alterations in brain regions and networks important for cognitive and affective processing have also been reported in VPT samples across the lifespan and have been studied among the possible biological mechanisms underlying the behavioural difficulties associated with VPT birth.^5-12^ It is important to highlight, however, that not only have previous studies identified brain changes associated with behavioural difficulties in those born VPT but have also characterized neural adaptions that support domain-specific performance.^13-17^ These findings, therefore, indicate that the functional reorganization of the VPT brain has complex implications for outcomes, as it may reflect both risk and resilience to behavioural difficulties.

Further complicating the understanding of brain–behavioural relationships in VPT populations is the fact that those born preterm tend to exhibit heterogenous behavioural outcomes. Previous studies aiming to stratify this heterogeneity implemented latent profile analyses using behavioural measures from both preterm and full-term (FT) born children.^18-20^ Their results indicated that while those born preterm were more likely to present with psychiatric, cognitive or socio-emotional difficulties, some preterm children displayed distinct profiles characterized by fewer or no behavioural difficulties. Moreover, while FT children predominantly exhibited more normo-typical behavioural profiles, some FT children displayed behavioural difficulties similar to those observed in preterm children.^18-20^ Together, these findings indicate that VPT and FT groups exhibit both within- and between-group heterogeneity, which needs to be addressed in order to develop individually tailored and biologically specific interventions aimed at supporting healthy development.^21,22^ This can be achieved by, first, implementing data-driven stratification approaches to identify distinct subgroups of individuals exhibiting similar behavioural profiles, irrespective of their birth status and, second, by investigating brain correlates differentiating between the distinct data-driven behavioural subgroups.

Similarly, individuals belonging to distinct diagnostic and non-diagnostic psychiatric groups also exhibit within- and between-group heterogeneity in terms of phenotypic profiles. Recent studies in psychiatric samples have successfully identified patterns of structural and functional connectivity characterizing distinct data-driven behavioural subgroups irrespective of diagnostic labels.^23-28^ A small number of studies in VPT children followed similar methodological approaches and investigated the underlying brain changes differentiating within-group behavioural heterogeneity. Results of these studies showed that early brain insult^29,30^ and structural and functional brain alterations^20,31^ characterized the distinct subgroups. However, it remains to be explored whether the heterogeneity in behavioural outcomes seen within and between VPT and FT born individuals persists into adulthood, and if it does, whether resting state functional connectivity (rsFC) changes may be associated with distinct data-driven behavioural phenotypes, irrespective of gestational age at birth.

Our study first aimed to identify long-lasting neurodevelopmental alterations associated with VPT birth, by investigating differences in rsFC and behavioural outcomes between VPT and FT born adults. Second, it aimed to delineate behavioural heterogeneity in VPT and FT born adults irrespective of gestational age at birth, by using a robust data-driven consensus clustering approach to stratify participants based on behavioural measures (executive function, attention, intelligence, socio-emotional processing, psychopathology and autistic traits) and to explore whether resultant data-driven behavioural subgroups would exhibit differences in rsFC. Finally, to address both within- and between-group heterogeneity, *post hoc* analyses investigated two-way group interactions between clinical (i.e. VPT versus FT birth) and behavioural (i.e. data-driven subgrouping) classification labels, to explore whether rsFC pattern and behavioural measure differences between VPT and FT adults varied according to distinct behavioural outcomes.

## Materials and methods

### Study design

#### Participants

VPT infants (i.e. born at <33 weeks of gestation) were recruited at birth from the Neonatal Unit at University College London Hospital (London, UK) between 1979 and 1985. Enrolled participants received cranial ultrasonographic imaging several times during the first week of life and weekly until discharge from hospital^32^ and were subsequently followed-up in childhood at 1, 4 and 8 years of age,^33,34^ adolescence (15 years), early (20 years) and adulthood (30 years).^35^ Age-matched controls, born at FT (37–42 weeks of gestation), were recruited from the community in middle adulthood. Exclusion criteria for the controls were any clinical complications at birth (i.e. prolonged gestation at >42 weeks, low birth weight <2500 g and receiving endotracheal mechanical ventilation). Exclusion criteria for both VPT and FT participants included severe hearing and motor impairments or history of neurological complications (i.e. meningitis, head injury and cerebral infections). For this study, we used magnetic resonance imaging (MRI) and behavioural data from the middle adulthood follow-up. Please see Supplementary Fig. 1 for more information about participants’ selection.

Research study practices were conducted in accordance with the Declaration of Helsinki. Ethical approval was granted by the South London and Maudsley Research and Ethics Committee and the Psychiatry, Nursing and Midwifery Research Ethics Subcommittee (PNM/12/13-10), King’s College London. All participants were native English speakers. Written informed consent was obtained from all study participants, and participant privacy rights were observed.

#### Clinical and socio-demographic details

Gestational age at birth and birth weight were collected from medical discharge notes for VPT participants. Participants born VPT were classified into three groups, according to cranial ultrasound diagnosis: no evidence of perinatal brain injury (no injury), Grade I–II peri-ventricular haemorrhage without ventricular dilation (minor injury) and Grade III–IV peri-ventricular haemorrhage with ventricular dilation (major injury).^36^

For both VPT and FT groups, self-reported ethnicity was recorded according to the following groups: African, Afro-Caribbean, Caucasian/White, Indian sub-continent and other. Socio-economic status was defined according to participants’ self-reported occupation and parental occupation at the time of the study. Occupations were categorized according to the Office of National Statistics, 1980 Standard Occupation Classification: I: higher managerial, administrative and professional occupations; II: intermediate occupations, small employers and own account workers and III: routine and manual occupations—lower supervisory and technical and semi-routine and routine occupations.

#### Cognitive assessments

The following cognitive assessments were administered to measure language, executive attention and general intelligence: Hayling Sentence Completion Test^37^; Controlled Oral Word Association Test (COWAT-FAS)^38^; four sub-tests from the Cambridge Neurophysiological Test Automated Battery (CANTAB) 2003 Eclipse version^39^: (i) Stockings of Cambridge (SOC), (ii) Intra-Extra Dimensional Set Shift (IED), (iii) Paired Associates Learning and (iv) Motor Screening Task (MOT); the Trail Making Task-B (TMT-B)^40^; Continuous Performance Test (CPT)—2nd edition^41^ and Wechsler Abbreviated Scale of Intelligence (WASI).^42^ Specific tasks are detailed in Supplementary Table 1.

#### Psychiatric and behavioural assessments

General psychopathology was measured using the Comprehensive Assessment of At-Risk Mental States (CAARMS),^43^ a semi-structured clinical interview, which measures aspects of psychopathology relating to mania, depression, suicidality and self-harm, mood swings/lability, anxiety, obsessive compulsive disorder symptoms, dissociative symptoms and impaired tolerance to normal stress; scores on the general psychopathology sub-scale were used in our analyses. The self-administered General Health Questionnaire (GHQ-12)^44^ was used to measure general well-being, Peters Delusion Inventory (PDI)^45^ to measure delusional ideation traits, Autism Quotient (AQ-10)^46,47^ to measure autism traits (i.e. social interaction, communication, attention switching, attention to detail and imagination), Social Adjustment Scale^48^ to measure participants’ satisfaction with their social situation and Role Functioning Scale^49^ to measure participants’ ability to function in their daily life. The Emotion Recognition Task (ERT)^50^ was administered to measure participants’ ability to recognize expressed emotions (happiness, sadness, surprise, anger, disgust and fear), as described in our previous work.^8^

#### Structural and functional MRI acquisition

MRI data were acquired at the Maudsley Hospital, London, UK, using a 3 Tesla Signa MR scanner (General Electric Healthcare). Structural fast spoiled gradient-echo (FSPGR) pulse sequence T1-weighted (T1w) images were collected using the following sequence parameters: repetition time (TR) = 7.1 ms, echo time (TE) = 2.8 ms, matrix = 256 × 256 and voxel size = 1.1 mm isotropic. Gradient-echo planar imaging resting state functional MRI data were collected while participants stared at a central cross on a screen for 8 min 32 s, using the following parameters: 256 volumes, TR = 2000ms, TE = 30 ms, flip angle = 75 degrees, matrix = 64 × 64, 37 non-contiguous slices of 2.4 mm thickness, 1.1 mm inter-slice gap and 3.4 mm in-plane resolution.

### MRI data pre-processing

Resting state functional MRI data pre-processing were performed using fMRIPrep 20.1.1, RRID:SCR_016216,^51^ which is based on Nipype 1.5.0, RRID:SCR_002502.^52^ In summary, steps included skull stripping, slice time correction, co-registration to the T1w reference image using boundary-based registration^53^ and head motion estimation (i.e. global signal and six motion parameters: three translation and three rotation parameters). The complete pre-processing protocol is detailed in the Supplementary material.

After pre-processing, data were de-noised by regressing out estimated motion confounders (i.e. global signal and six motion parameters: three translation and three rotation parameters) using the FMRIB Software Library *fsl_regfilt* command.^54^ A band-pass filter (0.01–0.1 Hz) was applied to the data using the AFNI software *3dBandpass* command.^55^ Participants were excluded if they exhibited excessive in-scanner head motion [i.e. mean frame-wise displacement (FD) exceeding 0.4 mm or a maximum FD exceeding 4 mm] or had functional MRI scans showing poor alignment with anatomical data. Sample sizes and participant exclusions are summarized in a flowchart in Supplementary Fig. 1.

### Brain parcellation and resting state functional connectivity estimation

Resting state functional MRI data were parcellated into bilaterally symmetric cortical regions using the Human Connectome Project Multi-Modal Parcellation (HCP-MMP) (v1) atlas^56^ and bilateral sub-cortical FreeSurfer regions.^57^ The two bilateral hippocampal regions from the HCP-MMP atlas were excluded as these regions were included as part of the FreeSurfer sub-cortical segmentation, resulting in a total of 374 regions included in our analyses (i.e. 358 HCP-MMP atlas bilateral cortical regions and 16 FreeSurfer bilateral sub-cortical regions).

An average of the functional MRI blood oxygen level–dependent signal time series across all voxels in each parcellation was used to estimate the regional time series for each of the 374 brain regions. For each participant, rsFC matrices were calculated using Pearson’s correlations between pairs of all 374 regional time series. A threshold of 0.2 was used to eliminate weak correlations (i.e. weights of edges with *r* ≥ 0.2 were retained) potentially corresponding to spurious connections.^58-60^ This was applied independently for each participant, followed by a Fisher *Z*-transformation.

### Consensus clustering

**Figure 1:**
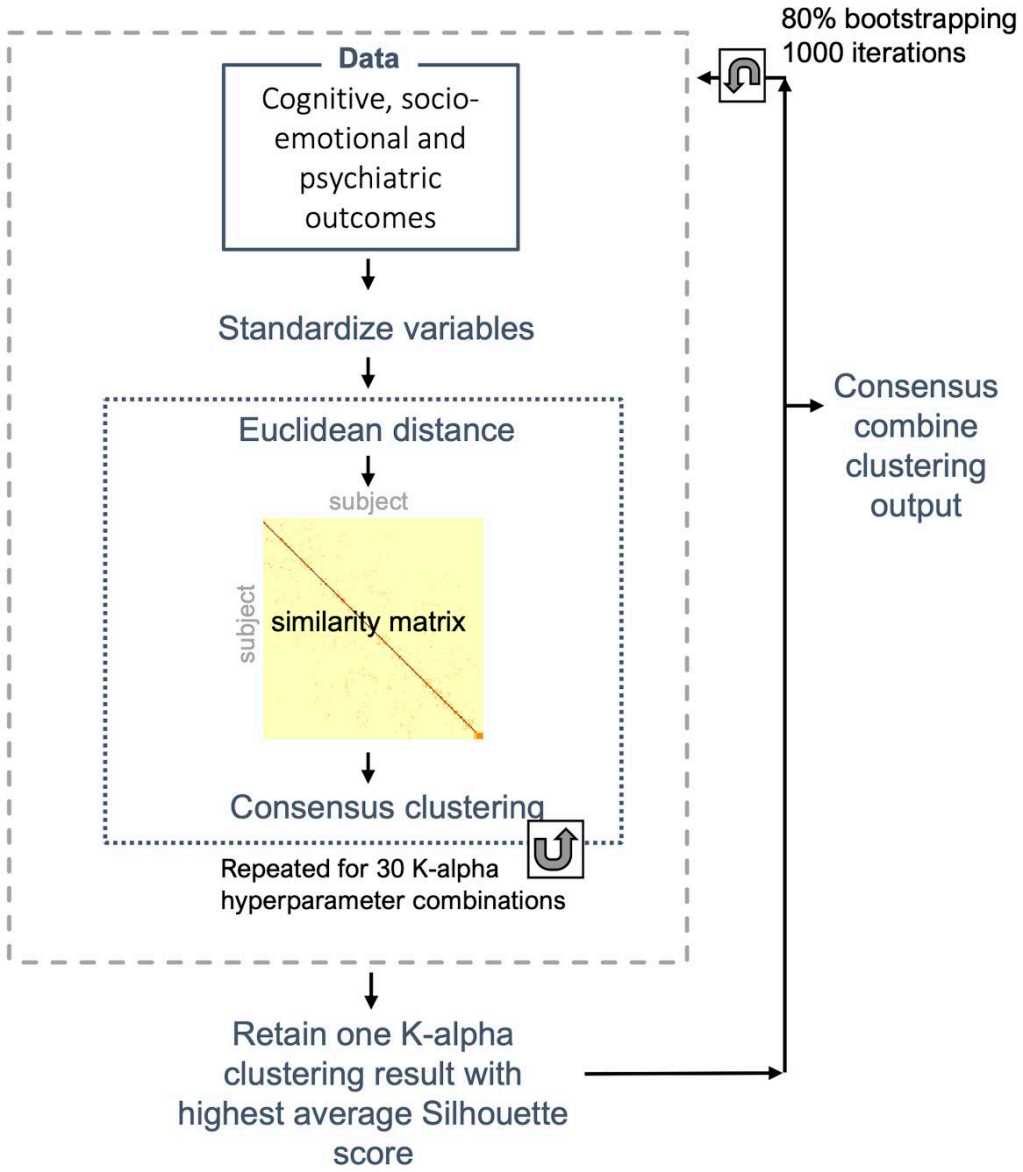
Consensus clustering pipeline followed.

To partition participants (both VPT and FT; *n* = 156) into data-driven behavioural subgroups, a consensus clustering pipeline (Fig. 1) was implemented using the following 13 behavioural measures as input features: COWAT-FAS mean total words produced, SOC total number of problems solved, IED total errors adjusted, MOT mean reaction time, TMT-B time elapsed, CPT total reaction time, full-scale IQ, total PDI score, total AQ-10 score, CAARMS total general psychopathology score, total GHQ score, ERT total number of correct responses and total SAS score (see Supplementary material for data pre-processing and feature selection procedures).

Each variable was first standardized to have a mean = 0 and standard deviation = 1, and an Euclidean distance matrix of the input data was calculated. A similarity matrix (network) was then calculated from the distance matrix, using the *affinityMatrix* function (SNFtool R package),^61^ which utilizes two hyper-parameters: neighbourhood size (*K*) and alpha (edge weighting parameter) that help increase the signal-to-noise ratio and in turn improve result validity and reliability. *K* corresponds to the number of surrounding nodes to consider for each node in the similarity network, and alpha determines a threshold for the strength of the edges in the similarity network (i.e. pairwise similarity between nodes within the sample). Greater *K* values result in more dense similarity networks and smaller values result in more sparse similarity networks, while greater alpha values result in weaker edges being retained and smaller alpha values result in similarity networks, which retain edges with higher similarity. Thirty different *K*-alpha combinations were used to generate 30 similarity networks based on the following values: *K* = 10, 15, 20, 25 and 30 and alpha = 0.3, 0.4, 0.5, 0.6, 0.7 and 0.8. These values lie within the ranges recommended in the SNFtool package: 10–30 for K and 0.3–0.8 alpha.^61^ Each of the resultant 30 similarity networks was successively inputted into the consensus clustering algorithm (*ConsensusClusterPlus* function, ConsensusClusterPlus R package),^62^ which performs agglomerative hierarchical clustering following a nested bootstrapping (*n* = 1000) spectral clustering for each of the 30 similarity networks. From the 30 resultant clustering outputs, the solution with the highest average silhouette width score was retained.

In order to improve the generalizability of our solution and avoid overfitting of hyper-parameter selection, the steps described in the above paragraph were repeated 1000 times where a randomized selection of 80% of the sample was used each time. The final resultant 1000 clustering outputs were then fed into a hierarchical clustering function (*consensus_combine,* DiceR package),^63^ to output a final consensus clustering result based on the consensus matrix.

To determine the optimal number of clusters (*C*), Eigengap and Rotation Cost metrics were first used to estimate the best and second-best number of clusters (*estimateNumberOfClustersGivenGraph* function SNFtool R package)^61^ for each of the 30 *K*-alpha combinations, identifying *C* = 2, *C* = 3 and *C* = 5 as the top three clustering solutions. We then ran the described consensus clustering pipeline three separate times, once for each of these solutions (*C* = 2, *C* = 3 and *C* = 5), and subsequently calculated consensus matrices and silhouette scores for each cluster solution. Resultant consensus matrix and silhouette score outputs suggested an optimal number of clusters of *C* = 2 (Supplementary Fig. 2); therefore, we evaluate subgroups obtained from the *C* = 2 solution.

The consensus clustering pipeline implemented here is adapted from the integrative clustering method used in our previous work,^31^ code: https://github.com/lailahadaya/preterm-ExecuteSNF.CC), where we do not apply the data-integration step in the current study.

### Statistical analyses

#### Evaluation of clinical, socio-demographic and behavioural profiles

The non-parametric Wilcox rank sum test was used for continuous variables and Chi-squared or Fisher’s Exact tests for categorical variables. Effect sizes were calculated using Wilcoxon Glass Rank Biserial Correlation for continuous variables and Cramer’s V (V) for categorical variables. False discovery rate (FDR) was used to account for multiple comparison testing.^64^ Sensitivity analyses using non-parametric permutation testing (5000 permutations) were adjusted for age, sex and socio-economic status.^65^  *P*-values of <0.05 were considered to be statistically significant.

#### Between-group differences in resting state functional connectivity at a topological network level

The Network Based Statistic (NBS), a cluster-based statistics approach, was applied.^66^ NBS implements the following steps: (i) mass univariate testing with a suitable statistical test of interest on all possible connections (i.e. edges), (ii) next, only edges with *P*-values below a pre-defined threshold (*P*-NBS-Threshold) are maintained, (iii) retained supra-threshold edges are then used to identify topologically connected structures (referred to as NBS ‘components’) present among the collection of supra-threshold edges using breadth-first search^67^ and finally, (iv) permutation testing is used to assign a Family Wise Error Rate–corrected *P*-value (*P*-FWER) for each identified component, based on the component’s strength. NBS testing is derived from traditional cluster-based thresholding of statistical maps; however, rather than generating clusters of voxels with spatial proximity in physical space, NBS can be applied to graph-like structures to generate clusters with inter-connected edges in topological space.^66,68^ An advantage of using NBS, compared with an approach that controls for FWER at an edgewise basis (such as FDR), is that it can provide increased statistical power by detecting the effect of interest in a collection of connections, which are collectively contributing to the effect of interest as opposed to uniquely contributing to the effect on an individual edgewise level.

Selecting a threshold in NBS [described in Step (ii) above] is a relatively arbitrary choice, which can be determined by experimenting with a selection of conservative and stringent thresholds.^66^ We ran NBS testing at three different *P*-value thresholds (i.e. *P*-NBS-Threshold = 0.05, 0.01 and 0.001) to identify relevant supra-threshold edges to be grouped into NBS components for further analysis. We implemented NBS testing with 1000 permutations using the NBR R package *nbr_lm* function (NBR).^69^ Statistical models tested included the following covariates: mean FD (as a measure of in-scanner head motion), sex, age and socio-economic status. The same sets of methods were implemented to identify differences in rsFC between (i) VPT and FT individuals and (ii) data-driven behavioural subgroups.

NBS generates two resultant outputs: (i) component strength or intensity—i.e. the sum of test statistic (*T*-statistic) values from all edges within the significant component and (ii) component size or extent—i.e. the number of connections comprising the significant component. We also calculated the number of connections belonging to each node within the component as a proportion of the total number of possible edges within that component and presented results graphically using the ggseg3d R package.^70^ To measure within- and between-network connectivity, we labelled nodes according to seven previously defined intrinsic connectivity networks [i.e. visual network, somatomotor network, dorsal attention network, ventral attention network (VAN), limbic network, frontoparietal network and default mode network (DMN)]^71^ and an eighth network comprised of 16 sub-cortical regions^72^ and calculated connectivity proportion and strength; the code is accessible at: https://github.com/frantisekvasa/functional_network_development/blob/master/nspn.fmri.R.

#### *Post hoc* exploratory analyses

We estimated the extent of nodal and edgewise overlap between the NBS components characterizing clinical (i.e. VPT versus FT birth) and data-driven behavioural subgrouping classifications using the Sørensen–Dice similarity coefficient, which is calculated as the ratio of two times the number of overlapping features between two sets, over the total number of features present across both sets,^73^ with values ranging between 0 and 1. The hyper-geometric cumulative density function was used to assess the significance of the overlapping edges between the two NBS components as described in the study by Tejavibulya *et al*.^74^

To address both within- and between-group heterogeneity, *post hoc* exploratory NBS analyses investigated whether differences in rsFC between VPT and FT clinical groups varied according to distinct behavioural outcome subgrouping, using two-way group interactions between clinical and data-driven behavioural classification labels. Two-way group interaction analyses were also used to investigate whether differences in behavioural outcomes between VPT and FT clinical groups varied according to behavioural outcome subgrouping.

We also investigated differences in early clinical risk (i.e. gestational age at birth, birth weight and peri-natal brain injury) and socio-demographic measures between VPT adults belonging to the distinct data-driven behavioural subgroups and in socio-demographic measures between FT adults in the distinct data-driven subgroups.

## Results

### Very preterm birth and full-term groups

**Figure 2:**
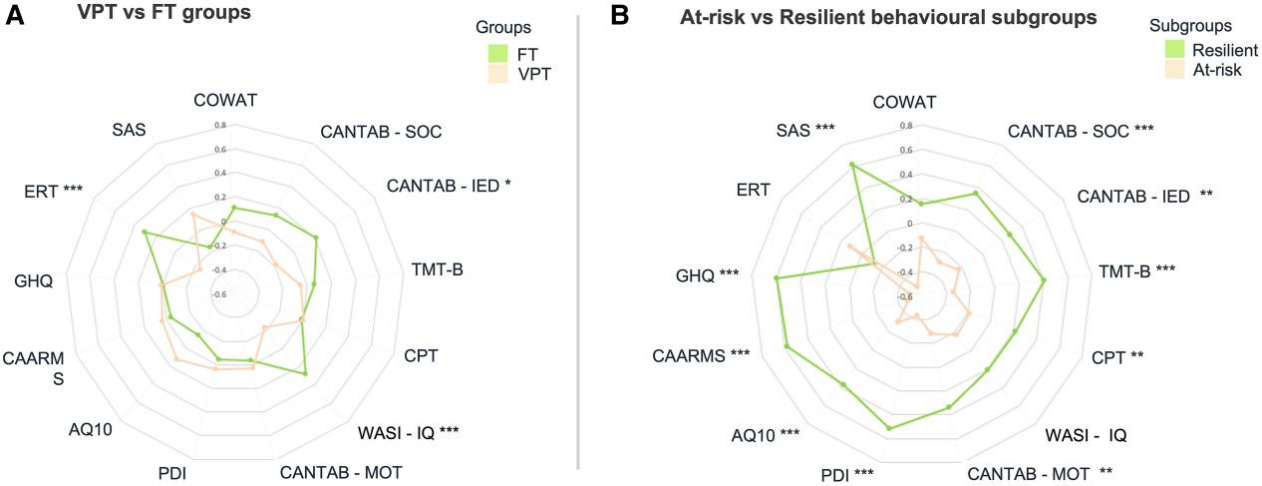
**Radar plots showing differences in behavioural profiles between (A) VPT and FT adults and (B) At-Risk and Resilient data-driven behavioural subgroups.**  *Z*-scores were computed for each group and plotted accordingly. For visual illustrative purposes, values for scales indicating poorer outcomes were reversed, so that larger *Z*-scores here indicate generally more optimal outcomes. *P < 0.05; **P < 0.01; ***P < 0.001. Statistical analyses investigating differences between groups were performed using the non-parametric Wilcox rank sum test.

The socio-demographic and clinical profiles of VPT and FT adults are summarized in Table 1, and their behavioural outcomes are given in Table 2 and Fig. 2A. In summary, adults born VPT had significantly lower full-scale IQ (WASI), attention set shifting (CANTAB-IED) and emotion recognition (ERT) scores than adults born (*P* < 0.05) FT. Head motion during functional MRI acquisition was greater in the VPT (median FD = 0.15 mm, range = 0.07–0.40 mm) than the FT group (median FD = 0.12 mm, range = 0.05–0.35 mm; *P* < 0.001). Supplementary analyses show that VPT adults excluded from analyses (*n* = 37) for reasons described in Supplementary Fig. 1 had relatively poorer cognitive and socio-emotional scores compared with those VPT adults included in the analyses (*n* = 116) (Supplementary Table 2).

**Table 1:** Clinical and socio-demographic characteristics of study participants used for the VPT versus FT analyses

	VPT (*N* = 116)	FT (*N* = 83)	*P*-value
Gestational age at birth, median (range) weeks	30.00 (24.00–32.00)	n/a	n/a
Birth weight, median (range) grams	1345 (552–2390)	3440 (2690–4990)	<0.001
Sex, *n* (%)			0.082
Male	66 (56.90)	36 (43.37)	
Female	50 (43.10)	47 (56.63)	
Ethnicity, *n* (%)^a^			0.139
African	2 (1.72)	5 (6.02)	
Afro-Caribbean	2 (1.72)	4 (4.82)	
Caucasian/White	76 (65.52)	55 (66.27)	
Indian sub-continent	8 (6.90)	2 (2.41)	
Other	4 (3.45)	6 (7.23)	
Peri-natal brain injury, *n* (%)^b^			n/a
No injury	62 (53.45)	n/a	
Minor injury	27 (23.28)	n/a	
Major injury	26 (22.41)	n/a	
Parental socio-economic status, *n* (%)^c^			0.220
I–II	43 (37.07)	38 (45.78)	
III	36 (31.04)	15 (18.07)	
IV–V	8 (6.90)	3 (3.62)	
Participants’ socio-economic status, *n* (%)^c^			<0.001
I–II	51 (43.97)	36 (43.37)	
III	41 (35.35)	26 (31.33)	
IV–V	6 (5.17)	0 (0.00)	
Student	1 (0.86)	16 (19.28)	
Unemployed	16 (13.8)	4 (4.82)	
Age at assessment, median (range) years	31.37 (23.346–39.33)	28.73 (26.26–36.49)	<0.001

Missing data: 29 VPT and 27 FT had missing parental socio-economic status; 1 VPT and 1 FT had missing participants’ socio-economic status data; 24 VPT and 11 FT had missing ethnicity data; 1 VPT has missing peri-natal brain injury classification.

n/a, not available.

^a^Ethnicity was self-reported.

^b^Ultrasound scans were used to classify peri-natal brain injury into three categories: no haemorrhage (no injury), Grade I–II peri-ventricular haemorrhage without ventricular dilation (minor injury) and Grade III–IV peri-ventricular haemorrhage with ventricular dilation (major injury).

^c^Socio-economic status was categorized according to the Office of National Statistics, 1980 occupation classifications: I: higher managerial, administrative and professional occupations; II: intermediate occupations, small employers and own account workers; III: routine and manual occupations—lower supervisory and technical and semi-routine and routine occupations.

**Table 2:** Behavioural outcomes in VPT and FT adults

	VPT (*n* = 116)	FT (*n* = 83)	*P*-value	FDR *P*-value	Adj. FDR *P*-value	Effect size
COWAT, total words^a^	13.00 (5.75)	14.00 (5.25)	0.052	0.166	0.115	−0.042
CANTAB—SOC, problems solved^a^	9.00 (2.75)	10.00 (2.00)	0.063	0.166	0.106	−0.064
CANTAB—IED, total errors adjusted^a^	15.00 (25.50)	10.50 (14.65)	0.002	0.007	0.021	0.184
TMT-B, time to finish task^a^	73.50 (40.50)	71.30 (39.05)	0.081	0.175	0.068	−0.093
CPT, total reaction time for correct responses^a^	417.50 (59.15)	414.00 (54.40)	0.921	0.921	0.936	−0.009
WASI—full-scale IQ^a^	106 0.00(13.75)	113.50 (12.25)	<0.001	<0.001	<0.001	0.088
CANTAB—MOT, reaction time^a^	691.00 (200.80)	734.00 (196.90)	0.307	0.399	0.456	0.062
PDI, total score^b^	21.50 (50.25)	18.00 (39.25)	0.406	0.480	0.530	0.002
AQ-10, total score^c^	2.00 (2.44)	3.00 (2.32)	0.198	0.322	0.257	0.121
CAARMS, general psychopathology score^d^	2.00 (5.50)	2.00 (4.00)	0.232	0.335	0.220	−0.111
GHQ, total score^e^	10.00 (6.00)	10.00 (7.00)	0.891	0.921	0.943	0.070
ERT, total correct^a^	56.60 (11.15)	62.00 (9.45)	**<**0.001	<0.001	<0.001	0.358
SAS, total score^a^	1.58 (0.45)	1.69 (0.53)	0.127	0.236	0.4021	0.136

Median (inter-quartile range) reported. ‘Adj. FDR *P*-value’ corresponds to the *P*-value after adjusting for covariates (sex, age and socio-economic status) and correcting for multiple comparisons with FDR. Effect sizes are calculated using Wilcoxon Glass Rank Biserial Correlation.

Missing data are as follows: ^a^FT *n* = 7, VPT *n* = 22; ^b^FT *n* = 21, VPT *n* = 22; ^c^FT *n* = 21, VPT *n* = 19; ^d^FT *n* = 12, VPT *n* = 17; ^e^FT *n* = 5, VPT *n* = 9.

### Data-driven behavioural subgroups

The socio-demographic and clinical profiles of VPT and FT study participants used for the data-driven consensus clustering analyses are summarized in Table 3. Two data-driven behavioural subgroups were identified and labelled as ‘At-Risk’ and ‘Resilient’, based on their observed phenotypic profiles (Table 4; Fig. 2B).

**Table 3:** Clinical and socio-demographic characteristics of study participants used for the clustering analyses

	VPT (*N* = 85)	FT (*N* = 71)	*P*-value
Gestational age at birth, median (range) weeks	30.00 (24.00–32.00)	n/a	n/a
Birth weight, median (range) grams	1317.50 (552.00–2390.00)		
Sex, *n* (%)			
Male	51 (60.00)	33 (46.48)	0.127
Female	34 (40.00)	38 (53.52)	
Ethnicity, *n* (%)^a^			0.127
African	2 (2.35)	5 (7.04)	
Afro-Caribbean	1 (1.18)	4 (5.63)	
Caucasian/White	70 (82.35)	53 (74.65)	
Indian sub-continent	7 (8.24)	2 (2.82)	
Other	3 (3.53)	5 (7.04)	
Perinatal brain injury, *n* (%)^b^			n/a
No injury	46 (54.12)	n/a	
Minor injury	19 (22.35)	n/a	
Major injury	19 (22.35)	n/a	
Parental socio-economic status, *n* (%)^c^			0.092
I–II	39 (45.88)	38 (53.52)	
III	32 (37.65)	14 (19.72)	
IV–V	8 (9.42)	3 (4.23)	
Participants’ socio-economic status, *n* (%)^c^			0.006
I–II	44 (51.77)	32 (45.07)	
III	29 (34.12)	23 (32.39)	
IV–V	2 (2.35)	0 (0.00)	
Student	1 (1.18)	11 (15.49)	
Unemployed	9 (10.59)	4 (5.63)	
Age at assessment, median (range) years	30.90 (26.25–35.48)	28.85 (24.21–39.33)	0.002

Missing data: 6 VPT and 16 FT had missing parental socio-economic status, 1 FT had missing participants’ socio-economic status data, 2 VPT and 2 FT had missing ethnicity data, 1 VPT has missing perinatal brain injury classification.

n/a, not available.

^a^Ethnicity was self-reported.

^b^Ultrasound scans were used to classify peri-natal brain injury into three categories: no haemorrhage (no injury), Grade I–II peri-ventricular haemorrhage without ventricular dilation (minor injury) and Grade III–IV peri-ventricular haemorrhage with ventricular dilation (major injury).

^c^Socio-economic status was categorized according to the Office of National Statistics, 1980 occupation classifications: I: higher managerial, administrative and professional occupations; II: intermediate occupations, small employers and own account workers; III: routine and manual occupations—lower supervisory and technical and semi-routine and routine occupations.

**Table 4:** At-Risk and Resilient behavioural subgroup profiles

	Subgroup 1—Resilient (*N* = 71)	Subgroup 2—At-Risk (*N* = 85)	*P*-value	FDR *P*-value	Adj. FDR *P*-value	Effect size
Age at assessment, years	29.83 (4.16)	30.22 (4.47)	0.972	0.972	n/a	−0.004
FD, mm	0.13 (0.07)	0.13 (0.06)	0.654	0.690	0.575	−0.042
COWAT, total words	14.00 (5.50)	13.00 (4.00)	0.071	0.097	0.117	0.168
CANTAB—SOC, problems solved	10.00 (2.00)	9.00 (2.00)	<0.001	<0.001	<0.001	0.371
CANTAB—IED, total errors adjusted	10.00 (11.00)	18.00 (26.60)	0.002	0.004	0.002	−0.289
TMT-B, time to finish task	61.00 (25.20)	78.00 (39.00)	<0.001	<0.001	<0.001	−0.428
CPT, total reaction time for correct responses	406.00 (51.30)	421.00 (61.40)	0.005	0.009	0.008	−0.260
WASI—full-scale IQ	112.00 (15.50)	108.00 (14.00)	0.038	0.059	0.008	0.194
CANTAB—MOT, reaction time	675.00 (171.50)	741.00 (255.00)	<0.001	<0.001	<0.001	−0.341
PDI, total score	13.00 (16.50)	41.80 (45.00)	<0.001	<0.001	<0.001	−0.596
AQ-10, total score	2.00 (1.92)	3.00 (2.71)	<0.001	<0.001	<0.001	−0.385
CAARMS, general psychopathology score	0.00 (2.00)	4.60 (4.20)	<0.001	<0.001	<0.001	−0.654
GHQ, total score	8.00 (2.00)	13.00 (6.00)	<0.001	<0.001	<0.001	−0.663
ERT, total correct	58.40 (12.60)	60.00 (9.00)	0.112	0.142	0.132	−0.148
SAS, total score	1.44 (0.26)	1.81 (0.50)	<0.001	<0.001	<0.001	−0.691
Birth status, *n* (%)			0.558	0.623	n/a	V = 0.060
VPT	41 (57.75)	44 (51.767)				
FT	30 (42.25)	41 (48.24)				
Sex, *n* (%)			0.169	0.200	n/a	V = 0.123
Male	43 (60.56)	41 (48.24)				
Female	28 (39.44)	44 (51.77)				
Participants’ socio-economic status, *n* (%)^a^			<0.001	0.001	n/a	V = 0.365
I–II	46 (64.79)	30 (35.29)				
III	21 (29.58)	31 (36.47)				
IV–V	0 (0.00)	2 (2.35)				
Student	1 (1.41)	11 (12.94)				
Unemployed	2 (2.82)	11 (12.94)				
Parental socio-economic status, *n* (%)^a^			0.055	0.080	n/a	V = 0.208
I–II	44 (61.97)	33 (38.82)				
III	16 (22.53)	30 (35.29)				
IV–V	5 (7.04)	6 (7.06)				

Median (inter-quartile range) reported unless stated otherwise where number of participants (*n*) is reported alongside percentage (%). ‘Adj. FDR *P*-value’ corresponds to the *P*-value after adjusting for covariates (sex, age and socio-economic status) and correcting for multiple comparisons with FDR. Effect sizes are calculated using Wilcoxon Glass Rank Biserial Correlation, unless otherwise stated. Cramer’s V (V) effect size was used for categorical variables.

n/a, not available.

^a^Socio-economic status was categorized according to the Office of National Statistics, 1980 occupation classifications; I: higher managerial, administrative and professional occupations; II: intermediate occupations, small employers and own account workers; III: routine and manual occupations—lower supervisory and technical and semi-routine and routine occupations.

In summary, the At-Risk subgroup had significantly less optimal executive function and attention scores probing spatial planning, attentional set shifting, visuo-motor coordination, comprehension abilities, sustained attention and response inhibition (CANTAB—SOC, MOT and IED, the TMT-B and CPT), compared with the Resilient subgroup (*P* < 0.05). The At-Risk subgroup also had significantly less optimal social adjustment, mental well-being and psychiatric scores (PDI, CAARMS, GHQ and SAS) and significantly increased autistic traits (AQ-10 scores), compared with the Resilient subgroup (*P* < 0.05). The two subgroups showed no differences in full-scale IQ (WASI), emotion recognition (ERT) or phonemic verbal fluency (COWAT). However, the At-Risk subgroup had a significantly higher proportion of individuals with lower own socio-economic status compared with the Resilient subgroup (*P* < 0.05). Parental socio-economic status did not differ between the subgroups.

**Figure 3:**
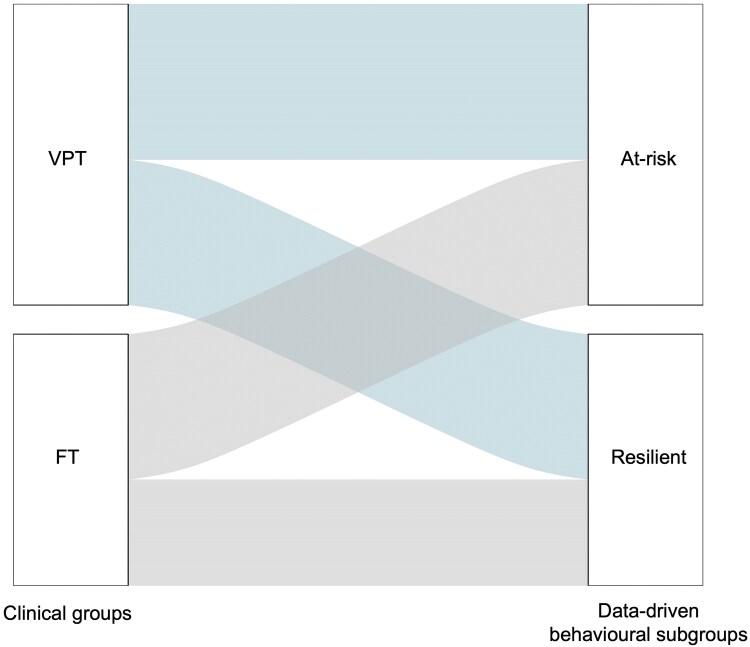
Alluvial plot showing VPT and FT adults clustering into the At-Risk and Resilient data-driven behavioural subgroups.

A total of 52% of the VPT adults in our sample clustered into the At-Risk subgroup and the remaining 48% into the Resilient subgroup (Fig. 3). Upon examining VPT adults only, there were no significant differences between the At-Risk and Resilient subgroups in terms of peri-natal clinical measures (i.e. gestational age, birth weight or perinatal brain injury) (Supplementary Table 3 and Fig. 3). In terms of parental socio-economic status, there were no differences between At-Risk and Resilient subgroups within VPT or FT adults (Supplementary Tables 3 and 4, respectively). As for participants’ own socio-economic status, only those born VPT displayed significant differences between the data-driven behavioural subgroups, where more VPT individuals with higher managerial, administrative and professional occupations belonged to the Resilient subgroup compared with the At-Risk subgroup (Supplementary Table 3) (*P* < 0.05). However, socio-economic status for those born FT did not differ significantly between the two data-driven subgroups (Supplementary Table 4).

*Post hoc* analyses investigating whether differences in behavioural outcomes between VPT and FT clinical groups varied according to behavioural outcome subgrouping report no significant two-way group interaction effects (Supplementary Table 5).

### Between-group differences in resting state functional connectivity

We report NBS analyses using *P*-NBS-Threshold values powered to detect a significant effect, while also reducing component size (i.e. not *P* = 0.05) (Supplementary Table 6). Main results reported here are from one-tailed NBS analyses using *P*-NBS-Threshold = 0.01, and additional sensitivity analyses investigating rsFC using a more stringent threshold (*P*-NBS-Threshold = 0.001) are reported in Supplementary Fig. 4.

#### Very preterm < full term

**Figure 4:**
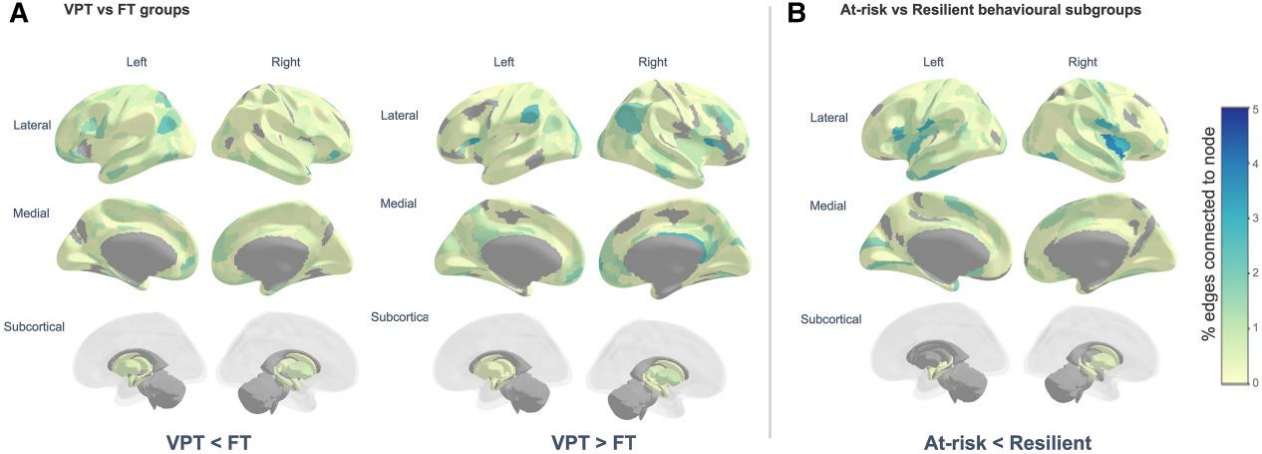
**Percentage of edges connected to each region (i.e. node) within the significant NBS components for (A) VPT versus FT groups and (B) At-Risk versus Resilient behavioural subgroups.** Darker colours denote higher percentages of edges, and lighter colours denote lower percentages, with areas marked in grey indicating regions that are not forming part of the NBS component. Statistical analyses investigating rsFC differences between groups were performed using NBS, which performed mass univariate linear models (correcting for covariates age, sex, in-scanner head motion and socio-economic status) on an edgewise level, with the following parameters: *P*-NBS-Threshold = 0.01 and 1000 permutations.

**Figure 5:**
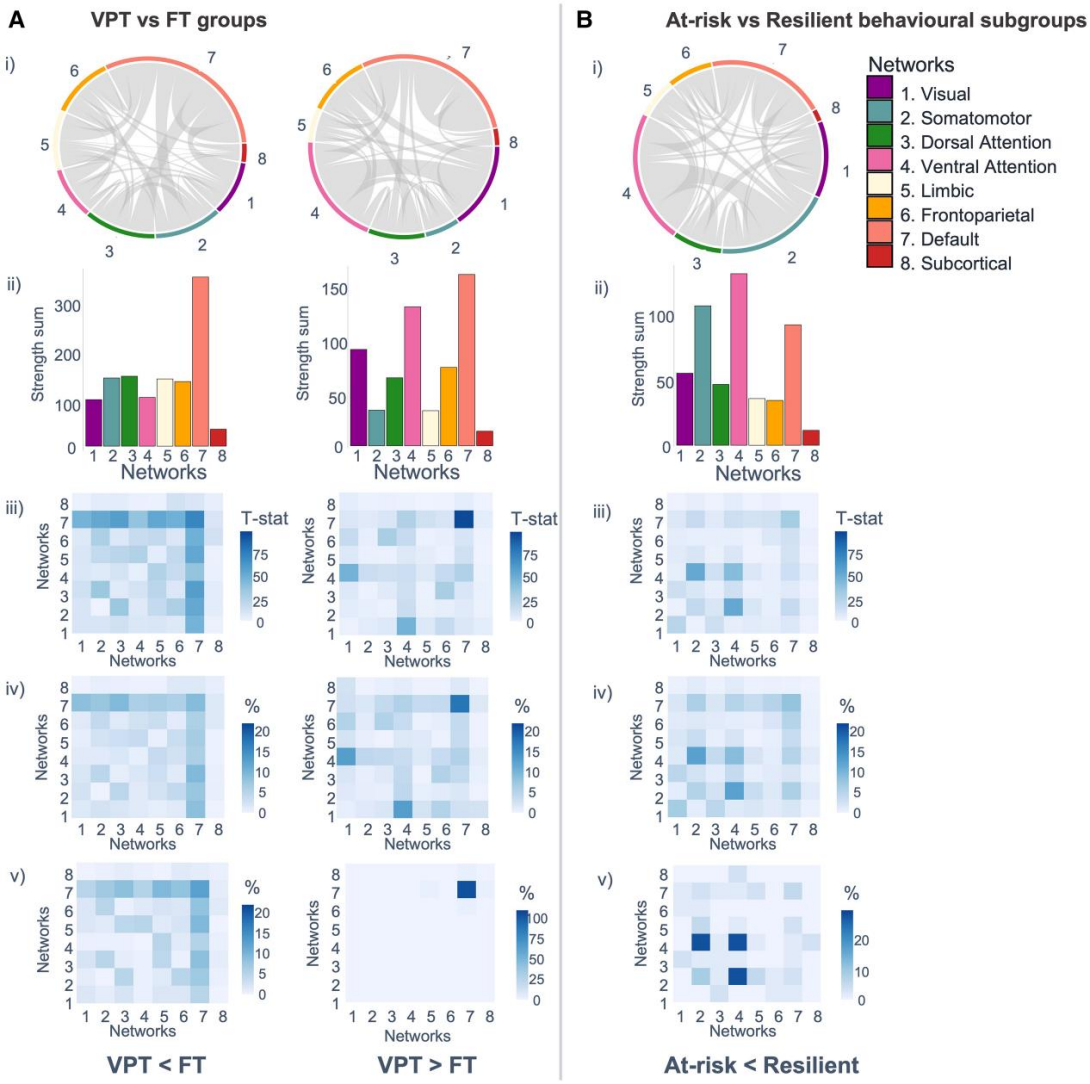
**Within- and between-network connectivity of the significant NBS components in (A) VPT versus FT groups and (B) At-Risk versus Resilient behavioural subgroups.** Results from main NBS analyses using a *P*-NBS-Threshold of 0.01: (i) circle plots illustrating within- and between-network connections within the significant component only, (ii) bar plots showing the sum of *T*-statistic strength values within the significant NBS component belonging to the different intrinsic connectivity networks (i.e. seven Yeo networks and an eighth network of sub-cortical regions) and (iii) within- and between-network connectivity strength (*T*-statistic sum). Heatmaps showing total number of within- and between-network connections as a percentage of the total number of connections forming the significant component: (iv) at *P*-NBS-Threshold = 0.01 and (v) *P*-NBS-Threshold = 0.001. Statistical analyses investigating rsFC differences between groups were performed using NBS, which performed mass univariate linear models (correcting for covariates age, sex, in-scanner head motion and socio-economic status) on an edgewise level.

NBS results showed weaker rsFC in the VPT group compared with the FT group (i.e. VPT < FT) in one component comprising 360 nodes (i.e. 96.25% of all regions) and 1467 edges (i.e. 2.10% of the 69 751 possible connections), with a component strength of 616.04 (*P*-FWER value = 0.007). Regions included in this component were widespread across the brain (Fig. 4A; Supplementary Table 7). Nodes with the highest number of connections within the component (i.e. component ‘hub’ regions) were predominantly localized to superior temporal gyrus, inferior and superior parietal cortex, inferior frontal, orbitofrontal, anterior cingulate and medial prefrontal cortex, inferior premotor, a lateral occipital/posterior temporal visual area, dorsolateral prefrontal cortex, medial and lateral temporal and posterior cingulate cortex. Component within- and between-network connectivity was highest in the DMN (Fig. 5A).

#### Very preterm > full term

NBS results also showed greater rsFC in the VPT group compared with the FT group (i.e. VPT > FT) in one component comprising 340 nodes (i.e. 90.91% of regions), 962 edges (i.e. 1.37% of possible connections) and component strength of 358.03 (*P*-FWER value < 0.001). ‘Hub’ regions within this component were less widespread across the brain and localized within posterior opercular cortex, posterior cingulate cortex, inferior parietal cortex, right orbitofrontal cortex, bilateral anterior cingulate and medial prefrontal cortex, superior temporal gyrus (auditory association cortex), dorsolateral prefrontal cortex, right lateral temporal cortex, right temporo-parietal-occipital junction and medial superior parietal cortex (Fig. 4A; Supplementary Table 8). The highest number of connections found in the component were within the DMN itself, followed by a moderate number of widespread connections in the VAN and especially between the VAN and the visual network.

A total of 326 nodes (i.e. 87.17% of regions) were present in both VPT < FT and VPT > FT components; however, the sets of edges connecting nodes within each component were mutually exclusive with no overlapping edges.

#### At-risk < resilient

Contrasts testing for lower rsFC in the At-Risk compared with the Resilient subgroup identified one significant NBS component with 337 nodes (i.e. 90.11% of regions), 832 edges (i.e. 1.19% of possible connections) and a strength sum of 309.04 (*P*-FWER = 0.019). Hub regions with the highest number of connections within the component were predominantly located in insular, frontal opercular and posterior opercular cortex (Fig. 4B; Supplementary Table 9). Other hub regions were found in the left inferior frontal cortex, lateral temporal cortex, right temporo-occipital visual area, left temporo-parieto-occipital junction, anterior cingulate, medial prefrontal cortex, left supplementary motor area, primary somatosensory cortex and the superior temporal sulcus (auditory association cortex) (Fig. 4B; Supplementary Table 9). Components within- and between-network connectivity were most pronounced between the VAN and somatomotor networks and within the VAN (Fig. 5B).

#### At-risk > resilient

No significant NBS components were detected when testing for higher rsFC in the At-Risk compared with the Resilient subgroup.

Confirming the robustness of the observed effects from analyses using a *P*-NBS-Threshold of 0.01, sensitivity NBS analyses using a more stringent *P*-NBS-Threshold of 0.001 reported significant components with greater sparsity (Supplementary Table 10) but largely similar rsFC patterns [Figs. 4, 5A(v) and 5B(v)].

*Post hoc* analyses investigating the interaction between clinical (VPT versus FT) groups and data-driven behavioural subgroups (At-Risk versus Resilient) on rsFC did not identify significant components (*P*-FWER > 0.05) at any *P*-NBS-Threshold examined (0.05, 0.01 and 0.001). Similarity index calculations indicated that the At-Risk < Resilient component had a high number of nodes, which were also part of the VPT < FT component (*n* = 325; Sørensen–Dice = 0.93) and the VPT > FT component (*n* = 304; Sørensen–Dice = 0.90), but very few edges overlapped with either clinical component: *n* = 9 edges (Sørensen–Dice = 0.01) and *n* = 22 edges (Sørensen–Dice = 0.03), respectively. Hyper-geometric cumulative density function calculations indicate no statistical significance of the observed overlapping edges (*P*-value = 0.811).

## Discussion

In this study, we compared rsFC between groups of adults stratified in terms of (i) their clinical characteristics (i.e. VPT and FT birth) and (ii) their behavioural profiles, identified using data-driven consensus clustering, regardless of their gestational age at birth. In VPT compared with FT adults, we identified complex preterm–specific patterns of both increased and decreased intrinsic rsFC predominately characterized by hypo-connectivity between the DMN and other networks examined and hyper-connectivity within the DMN and between the VAN and the visual network. When VPT and FT born adults were stratified in terms of their data-driven behavioural profiles, irrespective of gestational age at birth, we showed that an ‘At-Risk’ subgroup was characterized by more behavioural difficulties and reduced rsFC anchored in frontal opercular and insular areas of the VAN, relative to a ‘Resilient’ subgroup, characterized by more favourable behavioural outcomes.

In summary, our results indicate that there are complex and widespread long-lasting preterm–specific rsFC alterations, which we speculate may confer both risk and resilience to the behavioural sequelae associated with VPT birth. That is, while these rsFC alterations may partly explain the behavioural difficulties specific to those born VPT in cognitive and socio-emotional processing observed here, they may also aid the preservation of optimal outcomes in other behavioural domains where no between-group differences were noted (e.g. psychiatric difficulties, sustained attention, planning or phonemic verbal fluency). On the other hand, localized functional hypo-connectivity anchored in insular and frontal opercular regions observed in our study may characterize participants with unfavourable, compared with favourable, cognitive and behavioural outcomes, irrespective of birth status.

### Differences in resting state functional connectivity and behavioural outcomes between very preterm and full-term born adults

We identified complex patterns of both hypo- and hyper-connectivity predominantly located in the DMN, VAN and visual network in VPT compared with FT participants. Such rsFC alterations are evident in adulthood and may represent the neurobiological architecture underlying the attentional, cognitive and socio-emotional processing difficulties associated with VPT birth, commonly referred to as the ‘preterm behavioural phenotype’.^3^ However, in our cohort, VPT relative to FT born adults only differed in selected dimensions that have been studied as part of the ‘preterm behavioural phenotype’; they had lower full-scale IQ, difficulties in rule learning, attentional set shifting abilities (measured by the CANTAB-IED) and emotion recognition.

VPT adults, compared with FT controls, displayed functional hypo-connectivity between the DMN and the visual, somatomotor, dorsal attention, limbic and frontoparietal networks, as well as hyper-connectivity within the DMN itself. In line with our findings, patterns of both hyper- and hypo-connectivity in the DMN have been previously reported in VPT born children and adults,^5,75-77^ suggesting that functional DMN connectivity alterations may characterize VPT samples. Functional DMN connectivity emerges during the third trimester of gestation, a critical period of brain development during which VPT infants are born, and previous studies have reported structural and functional brain alterations at term-equivalent age in regions belonging to the DMN.^78-82^ Extending beyond preterm populations, functional alterations in the DMN have been described in several psychiatric conditions, including schizophrenia, anxiety and mood disorders,^83,84^ suggesting that the DMN rsFC alterations observed in VPT individuals may represent neurobiological alterations, which could contribute to the behavioural difficulties associated with VPT birth.

On the other hand, alterations in DMN rsFC have also been studied as adaptive neural mechanisms; for instance, maintaining attentional capture (i.e. less distractibility) in male veterans.^85^ Such findings suggest that functional reorganization of the DMN may also reflect compensatory biological alterations supporting selective cognitive and behavioural processing in VPT individuals; in this context, referring to the behavioural outcomes where no between-group differences were noted in our study sample, including spatial planning (CANTAB-SOC), coordination (MOT), cognitive flexibility (TMT-B), phonemic verbal fluency (COWAT), sustained attention (CPT), social adaptation (SAS), prodromal symptoms (PDI), autism traits (AQ-10) and general psychopathology (CAARMS and GHQ). This finding emphasizes the notion that complex neurobiological alterations following VPT birth may confer both risk and resilience to the long-term consequences of VPT birth. Further supporting this point, we also identified patterns of hyper-connectivity in the VPT relative to the FT group in the VAN, a ‘circuit-breaker’ network that disengages during tasks requiring focused attention and activates to redirect attention towards external task-irrelevant stimuli.^86,87^ Notably, the highest proportion of connections were between the VAN and the visual network, which may reflect adaptive functional reorganization in the VPT group. In a previous study, stronger rsFC changes in visual and attention networks were associated with fewer attention deficits in visual short-term memory storage in VPT relative to FT adults.^14^ Another study found that attention processing was selectively supported by VAN and visual network connectivity in VPT born children and by dorsal attention, frontoparietal and cingulo-opercular network connectivity in FT controls.^77^ The authors argued that VPT children may have a greater reliance on visually stimulated ‘bottom-up’ neural processes to maintain attention mechanisms, which is in line with their previous findings showing poorer attention abilities in VPT children with reduced volumes in regions of the visual network.^88^

We also identified that component ‘hub’ regions (i.e. those with a high percentage of connections within the component) with higher rsFC in the VPT group relative to the FT group were localized to brain regions previously identified as nodes of a ‘rich-club’ network (i.e. the sub-network of highly connected brain regions, which are also highly connected to one another), important for efficient integration and transfer of information between systems.^89,90^ We previously reported stronger rich-club network structural connectivity and weaker peripheral connectivity in an overlapping sample of VPT adults compared with FT controls and argued that increased resources in the VPT brain may be preferentially allocated to the rich-club network in order to maintain efficient information exchange across the brain.^91^ Furthermore, overlapping areas in higher order association cortices seem to have the greatest levels of inter-individual variability in adulthood^92^ and preterm neonates at term.^93^ They have also been reported to demonstrate the greatest increase in variability from infancy to adulthood in preterm born individuals,^93^ suggesting long-lasting opportunity for environmental post-natal factors to contribute towards the development of adaptive neural mechanisms in the VPT adult.

### Differences in resting state functional connectivity and behavioural outcomes between data-driven behavioural subgroups

Considering the neurodevelopmental heterogeneity exhibited within and between those born VPT and FT, it remains to be established whether rsFC may be useful to characterize the behavioural difficulties observed in VPT individuals.^2,94^ Aiming to address this question, we stratified VPT and FT adults into data-driven behavioural subgroups and investigated specific rsFC alterations which may differentiate them. We identified two data-driven behavioural subgroups, irrespective of birth status (VPT and FT): an ‘At-Risk’ subgroup with more executive function, attention, socio-emotional and psychiatric difficulties, compared with a ‘Resilient’ subgroup, with more favourable behavioural outcomes. Notably, the behavioural differences observed between data-driven subgroups were more pronounced than those observed between VPT and FT adults.

We also identified underlying rsFC differences characterizing the distinct data-driven behavioural subgroups, where the At-Risk, compared with the Resilient subgroup, displayed hypo-connectivity within the VAN and between the VAN and the somatomotor network. Specifically, the predominant connectivity patterns forming this component were anchored in frontal opercular and insular regions of the brain, which play an integral role in detecting bottom-up salient information from the environment and switching between networks to produce appropriate cognitive control, socio-emotional and interoceptive somatomotor responses.^95-100^ Our findings are in line with previous studies showing structural and functional alterations in insular and opercular regions in adults experiencing mental health difficulties^101,102^ and executive dysfunction.^103^ Furthermore, studies investigating rsFC across multiple psychiatric groups identified trans-diagnostic patterns of hypo-connectivity in lower-order networks, such as the somatomotor network, as well as higher order networks, such as the VAN.^104,105^ The rsFC patterns identified here characterized data-driven behavioural subgroups irrespective of gestational age at birth (VPT and FT), indicating that these specific neural mechanisms may represent biomarkers of behavioural outcomes in the general population, which are not unique to VPT individuals. We also found no significant interaction effects between birth group (VPT versus FT) and data-driven behavioural subgroups (At-Risk versus Resilient) on rsFC and very little overlap in rsFC between the clinical and behavioural components identified by NBS, which may further support our speculation that the differences in rsFC between the data-driven subgroups may be characterizing behavioural outcomes independently of gestational age at birth. However, future studies with larger samples, and hence greater statistical power, may further investigate the possible influence of VPT (versus FT) birth on the relationship between rsFC alterations and behavioural outcomes.

Our *post hoc* analyses aimed to explore whether specific enriching factors, or lack of certain social or clinical risk factors, protected the VPT adults belonging to the Resilient subgroup from developing an At-Risk behavioural profile. In contrast to previous studies in VPT children, we found that perinatal clinical risk was not higher in VPT adults who belonged to an At-Risk (versus Resilient) subgroup.^31,106^ Social risk, on the other hand, may be specifically related to the difficulties observed in the VPT At-Risk subgroup, which contained more VPT adults from more socially disadvantaged backgrounds compared witth the Resilient subgroup, while this relationship was not observed in FT adults. These findings and previous studies in children^20,30,31,107^ could be interpreted within a ‘differential susceptibility’ framework, which posits that vulnerable individuals (e.g. those born VPT) are particularly sensitive to environmental influences, where negative or positive factors [such as social (dis)advantage] can promote either worse or more optimal outcomes, respectively.^108^ Therefore, VPT adults in the At-Risk subgroup may have experienced a ‘double-hit’ of being born VPT and being socio-economically disadvantaged. Nonetheless, it is worth noting that socio-economic status in our sample only partially explained behavioural outcomes, as our main behavioural and rsFC results remained significant after adjusting for this covariate. It is therefore plausible that additional unmeasured environmental or hereditary factors (e.g. parental mental health or cognitively stimulating home environment)^20,31,107^ may have contributed to the behavioural outcomes observed in the distinct subgroups.

This study has several strengths, which include the use of a large sample of both VPT and FT born controls, the implementation of rigorous consensus clustering methods to obtain data-driven behavioural subgroups and the use of fMRIPrep, a robust automated resting state functional MRI pre-processing pipeline that promotes pre-processing transparency and aims to alleviate hurdles related to reproducibility in functional MRI analyses.^51,109^ We also acknowledge several limitations to our study. For instance, we recognize that the choice of 0.2 as the threshold to eliminate weak connections is relatively arbitrary. While some argue that thresholding has benefits in reducing the number of spurious connections assessed and hence enhances biological plausibility,^60^ others report no practical benefits from thresholding.^110^ Furthermore, after excluding participants with excessive head motion, behavioural outliers, missing data or poor alignment of functional MRI data, supplementary analyses showed that the sub-sample of VPT adults used in our analyses had relatively better cognitive and socio-emotional processing outcomes in comparison with VPT adults excluded from the analyses. This may limit the generalizability of our results to cohorts of low-risk VPT adults with relatively favourable behavioural outcomes. It may also explain why our two data-driven behavioural subgroups have similar proportions of VPT and FT born individuals, which is not in line with previous studies in children, which have reported higher ratios of VPT to FT individuals belonging to At-Risk subgroups and lower ratios to Resilient subgroups.^18,20^ On the other hand, our results may be reflective of the increased rates of mental health difficulties with increasing age, which may not yet be apparent in childhood.^111,112^ Future studies with more representative samples of VPT adults could help elucidate these potentially inconsistent findings. Furthermore, in our analyses, we did not account for structural brain changes, which we have previously reported between an overlapping sample of VPT and FT individuals.^35^ This represents a limitation of the current study, as brain anatomy necessarily constrains function^113^ and early brain injury has been associated with alterations of functional (and structural) connectivity in precharterm samples.^114^ Another limitation is the lack of availability of information about postnatal treatment and course and co-morbidities such as bronchopulmonary dysplasia, which are known to be associated with both behavioural outcomes and alterations in brain connectivity.^115-118^ Another possible limitation is that we did not include known risk factors (such as socio-economic status, parenting or clinical measures) in the clustering model, which may have increased the difficulty in identifying nuanced subgroups exhibiting ‘equifinal’ trajectories (i.e. those with similar behavioural outcomes but distinct underlying risk factors).^31,119^ The heterogeneity in underlying risk factors exhibited by those born VPT could also potentially hinder the ability to detect group differences between VPT and FT individuals. However, to our knowledge, this is the first study to parse behavioural heterogeneity in VPT adults; therefore, we decided to follow an approach similar to those implemented in the vast majority of studies in VPT children, where individual-level behavioural variables were included as inputs to the clustering model and risk factors were explored *post hoc*.^18-20,29,30,106,120^

In summary, this study shows that there are complex patterns of rsFC alterations, which are specifically associated with VPT birth in adult life. We speculate that these alterations may reflect neural adaptations conferring both risk and resilience to the long-term sequelae of VPT birth. We also identify distinct rsFC alterations in insular and frontal opercular regions in a data-driven At-Risk relative to a Resilient behavioural subgroup, irrespective of birth status (VPT versus FT), indicating that these neurobiological changes may reflect biomarkers of behavioural outcomes in the general population that are not unique to those born VPT.

## Supplementary Material

fcaf074_Supplementary_Data

## Data Availability

Access to data supporting the published work can be made available upon request from the corresponding author. Code used to label nodes according to intrinsic network membership is accessible here: https://github.com/frantisekvasa/functional_network_development/blob/master/nspn.fmri.R, and code used to run consensus clustering pipelines is adapted from code accessible here: https://github.com/lailahadaya/preterm-ExecuteSNF.CC.
