# Supplementary material for: Exploring functional connectivity in clinical and data-driven groups of preterm and term adults
Source: Brain Commun. 2025 Feb 17;7(2):fcaf074. doi: 10.1093/braincomms/fcaf074 (PMC11891483; doi:10.1093/braincomms/fcaf074)
Supplement: fcaf074_Supplementary_Data [file fcaf074_supplementary_data.pdf]

Supplementary Materials

Anatomical and functional MRI data pre-processing with fMRIPrep

The full description of the anatomical and functional data pre-processing pipeline below is extracted from the boilerplate automatically generated by fMRIPrep (released under the CC0 license).

Anatomical data pre-processing. The T1-weighted (T1w) image was corrected for intensity non-uniformity (INU) with N4BiasFieldCorrection [1], distributed with ANTs 2.2.0 (RRID:SCR_004757, [2]), and used as T1w-reference throughout the workflow. The T1w-reference was then skull-stripped with a Nipype implementation of the antsBrainExtraction.sh workflow (from ANTs), using OASIS30ANTs as target template. Brain tissue segmentation of cerebrospinal fluid (CSF), white-matter (WM) and gray-matter (GM) was performed on the brain-extracted T1w using fast (FSL 5.0.9, RRID:SCR_002823, [3]). Brain surfaces were reconstructed using recon-all (FreeSurfer 6.0.1, RRID:SCR_001847, [4]), and the brain mask estimated previously was refined with a custom variation of the method to reconcile ANTs-derived and FreeSurfer-derived segmentations of the cortical gray-matter of Mindboggle (RRID:SCR_002438, [5]). Volume-based spatial normalization to one standard space (MNI152NLin2009cAsym) was performed through nonlinear registration with antsRegistration (ANTs 2.2.0), using brain-extracted versions of both T1w reference and the T1w template. The following template was selected for spatial normalization: ICBM 152 Nonlinear Asymmetrical template version 2009c [[6], RRID:SCR_008796; TemplateFlow ID: MNI152NLin2009cAsym].

Functional data pre-processing. For each of the 1 BOLD runs found per subject (across all tasks and sessions), the following preprocessing was performed. First, a reference volume and its skull-stripped version were generated using a custom methodology of fMRIPrep. Head-motion parameters with respect to the BOLD reference (transformation matrices, and six corresponding rotation and translation parameters) are estimated before any spatiotemporal filtering using mcflirt (FSL 5.0.9, [7]. BOLD runs were slice-time corrected using 3dTshift from AFNI 20160207 (Cox and Hyde 1997, RRID:SCR_005927). Susceptibility distortion correction (SDC) was omitted. The BOLD reference was then co-registered to the T1w reference using bbregister (FreeSurfer) which implements boundary-based registration [8].

Co-registration was configured with six degrees of freedom. The BOLD time-series (including slice-timing correction when applied) were resampled onto their original, native space by applying the transforms to correct for head-motion. These resampled BOLD time-series will be referred to as preprocessed BOLD in original space, or just preprocessed BOLD. The BOLD time-series were resampled into standard space, generating a preprocessed BOLD run in MNI152NLin2009cAsym space. First, a reference volume and its skull-stripped version were generated using a custom methodology of fMRIPrep. Several confounding time-series were calculated based on the preprocessed BOLD: framewise displacement (FD), DVARS and three region-wise global signals. FD was computed using two formulations following Power (absolute sum of relative motions, [9]) and Jenkinson (relative root mean square displacement between affines, [7]). FD and DVARS are calculated for each functional run, both using their implementations in Nipype (following the definitions by [9]). The three global signals are extracted within the CSF, the WM, and the whole-brain masks. Additionally, a set of physiological regressors were extracted to allow for component-based noise correction (CompCor, [10]). Principal components are estimated after high-pass filtering the preprocessed BOLD time-series (using a discrete cosine filter with 128s cut-off) for the two CompCor variants: temporal (tCompCor) and anatomical (aCompCor). tCompCor components are then calculated from the top 5% variable voxels within a mask covering the subcortical regions. This subcortical mask is obtained by heavily eroding the brain mask, which ensures it does not include cortical GM regions. For aCompCor, components are calculated within the intersection of the aforementioned mask and the union of CSF and WM masks calculated in T1w space, after their projection to the native space of each functional run (using the inverse BOLD-to-T1w transformation). Components are also calculated separately within the WM and CSF masks. For each CompCor decomposition, the k components with the largest singular values are retained, such that the retained components time series are sufficient to explain 50 percent of variance across the nuisance mask (CSF, WM, combined, or temporal). The remaining components are dropped from consideration. The head-motion estimates calculated in the correction step were also placed within the corresponding confounds file. The confound time series derived from head motion estimates and global signals were expanded with the inclusion of temporal derivatives and quadratic terms for each [11]. Frames that exceeded a threshold of 0.5 mm FD or 1.5 standardised DVARS were annotated as motion outliers. All resamplings can be performed with a single interpolation step by composing all the pertinent transformations (i.e. head-motion transform matrices, susceptibility distortion correction when available, and co-registrations to anatomical and output spaces). Gridded (volumetric)

resamplings were performed using antsApplyTransforms (ANTs), configured with Lanczos interpolation to minimize the smoothing effects of other kernels [12]. Non-gridded (surface) resamplings were performed using mri_vol2surf (FreeSurfer).

Many internal operations of fMRIPrep use Nilearn 0.6.2 [13], RRID:SCR_001362), mostly within the functional processing workflow. For more details of the pipeline, see the section corresponding to workflows in fMRIPrep's documentation (https://fmriprep.org/en/latest/workflows.html).

Behavioural data pre-processing and consensus clustering feature selection

Behavioural variables with data missing in >25% of the sample (i.e., total RFS scores) or with outlier data point values in >5% of the sample (i.e., PAL task total adjusted errors score and HSCT total scores) were excluded from the analyses. Participants were excluded from the analyses if they had more than 25% of behavioural data missing or had outlier data points on any of the included behavioural measures. Any remaining missing data were imputed using the K-nearest neighbour method. The final sample used in the consensus clustering pipeline excluded participants not included in the functional connectivity analysis and those with further outlier data points on any of the included behavioural measures (Supplementary Fig. 1). Outliers are defined as data points with values exceeding the median by three times the interquartile range or more.

Supplementary figures

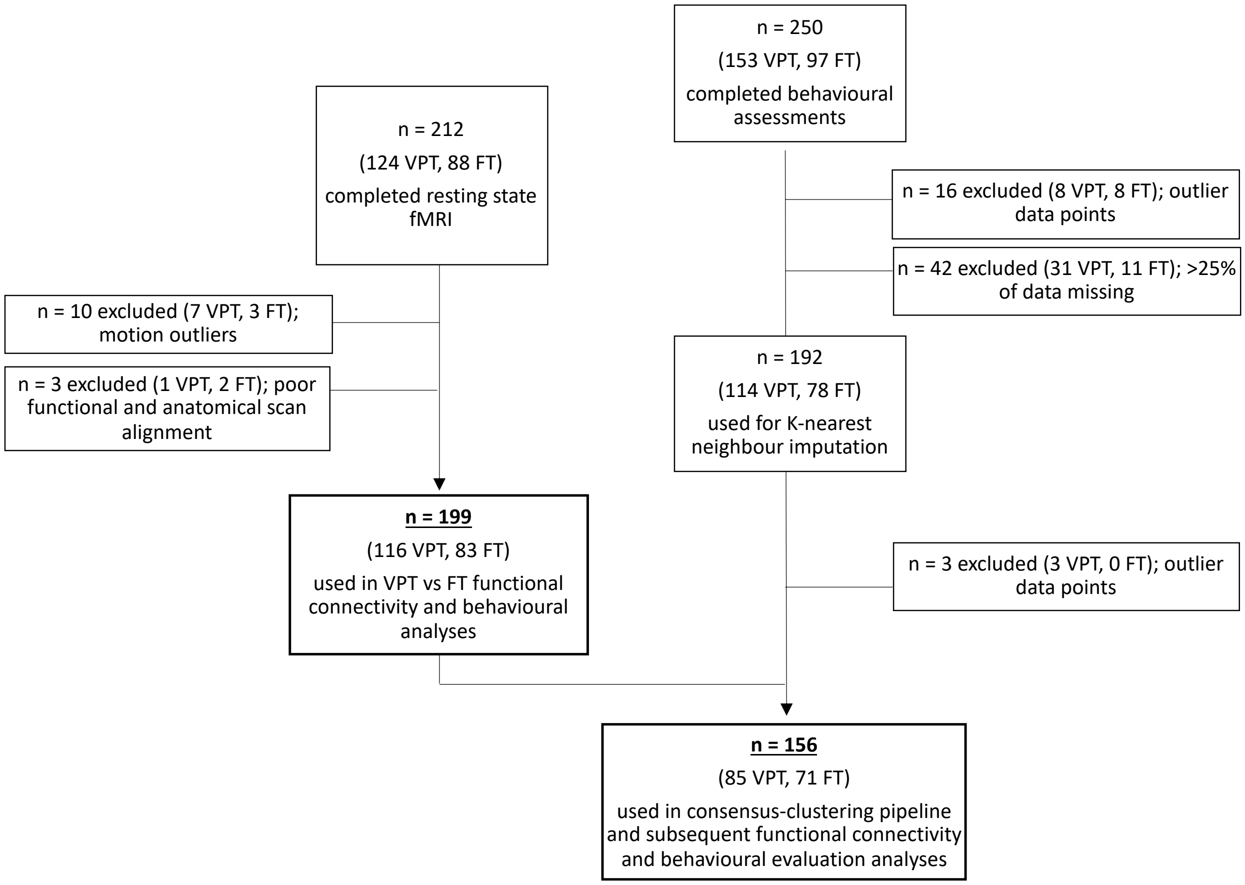

Supplementary Figure 1. Participants' selection flow diagram.

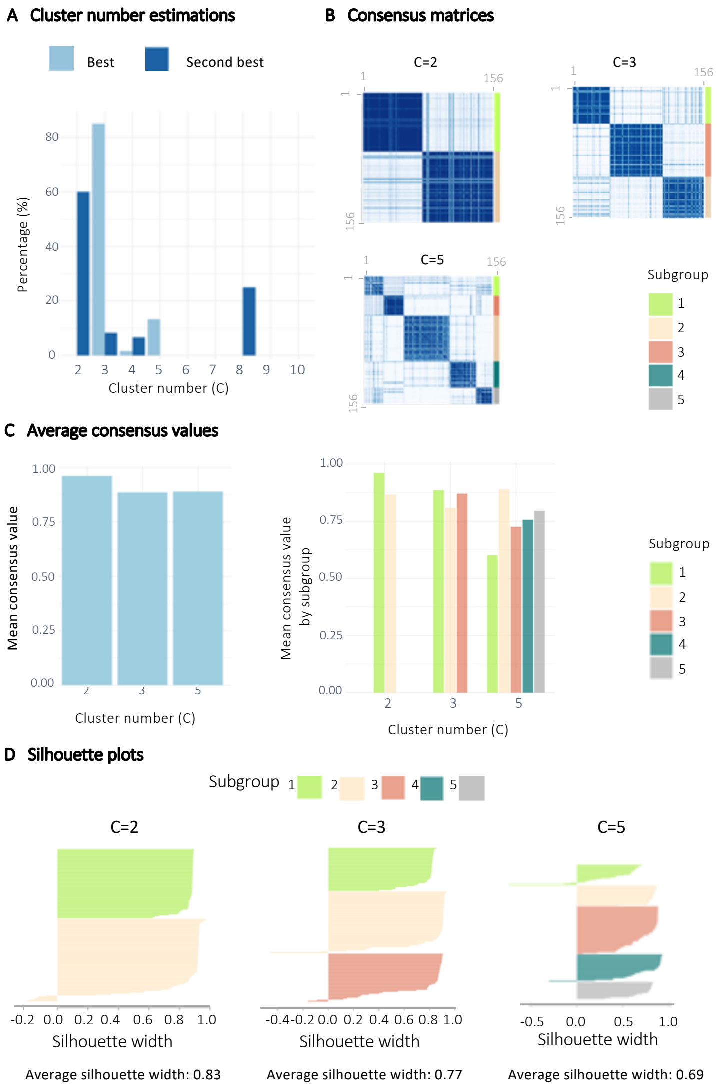

Supplementary Figure 2. Estimating the optimal number of clusters. A) Number of times (in percent) each number of clusters was selected as the best (pale blue) or second best (blue) using Eigengap and Rotation Cost for the 30 combinations of K-alpha parameters, with C=2 and C=3 being the most frequently estimated best and second best number of clusters, followed by C=5. B) Consensus matrices from C=2, C=3 and C=5 showing consensus values (i.e., proportion of times each pair of subjects co-clustered into the same cluster over the 1,000 iterations; darker blue indicates higher proportions of co-clustering. C) Mean consensus values for each number of clusters (C=2, C=3 and C=5) and for each subgroup within the different number of clusters runs (left and right, respectively) with the highest values belonging to C=2. D) Silhouette width values for each subgroup within the different number of clusters runs: C=2, C=3 and C=5, whereby C=2 was also displaying the highest values.

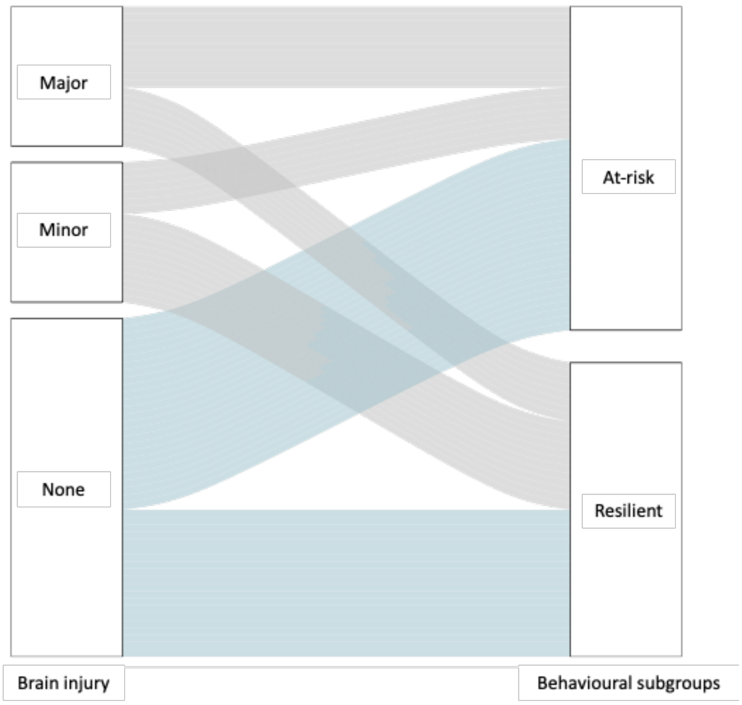

Supplementary Figure 3. Alluvial plots showing VPT individuals with no brain injury (in blue) and minor or major brain injury (in grey) clustering into the At-risk and Resilient data-driven behavioural subgroups.

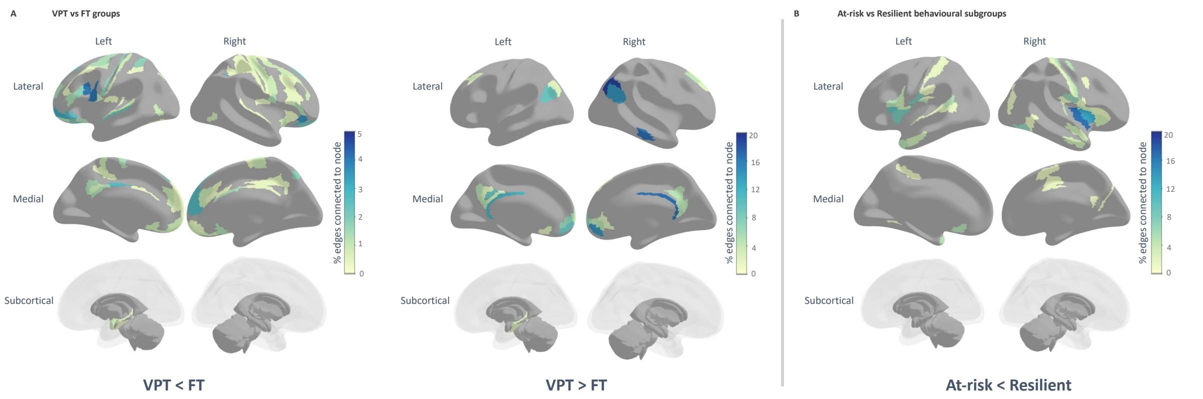

Supplementary Figure 4. Percentage of edges connected to each region within the significant NBS components at 0.001 p-NBS-Threshold. Darker colours (blue) denote higher percentages and lighter colours (yellow) denote lower percentages, with areas marked in grey indicating regions that are not forming part of the NBS component. Statistical analyses investigating rsFC differences between groups were performed using NBS, which performed mass-univariate linear models (correcting for covariates age, sex, in-scanner head motion, and socio-economic status) on an edgewise level, with the following parameters: p-NBS-Threshold = 0.001 and 1000 permutations.

Supplementary tables

Supplementary Table 1. Cognitive assessment descriptions.

Assessment	Assessment description	Construct measured by assessment
**Hayling Sentence Completion Test (HSCT)**	Participants are asked to complete sentences correctly and incorrectly by providing semantically related or unrelated words.	This task **measures both response initiation speed and response inhibition**. Total scaled score is measured using response latency and response errors.
**Controlled Oral Word Association Test (COWAT-FAS)**	Participants are given 60 seconds to list as many words (excluding proper names, numbers, or words with different tenses/endings) as possible beginning with a given letter (i.e., F, A, and S).	This task **measures phonemic verbal fluency**. The total score of phonemic fluency is calculated as the sum of total words produced for each letter.
**Stockings of Cambridge (SOC) task**	Participants are required to rearrange three coloured stimuli to match the pattern of the stimuli displayed on the screen in the minimum number of moves possible.	This task **measures spatial planning abilities**. A score of 'Problems Solved in Minimum Moves' is calculated.
**Intra-Extra Dimensional Set Shift (IED) task**	Participants are presented with stimuli and are required to learn a rule and select correct responses. The task involves categorising visual stimuli into sets (i.e., the visual discrimination of shapes vs lines) and being able to flexibly respond to changes in stimuli (i.e., shifting attention). The rule changes are intra-dimensional at first (i.e., the shapes or lines are still the relevant stimuli) and then become extra-dimensional (i.e., the shapes or lines are no longer the relevant stimuli).	This task **measures attentional set shifting**. The number of errors made are calculated and adjusted for task stages completed by adding errors for stages not completed. Outcome measure is a 'Total Errors Adjusted' score.
**Paired Associates Learning (PAL) task**	Multiple patterns appear on a screen in a random order and then disappear. These patterns reappear, and the participant is asked to recall where on the screen that pattern was originally displayed.	**Measures episodic visuo-spatial associative memory**. The total number of adjusted errors is used a summary score.
**Motor Screening Task (MOT)**	A cross appears on the screen and participants are asked to press on it as quickly and as accurately as possible.	This task **measures visuo-motor coordination and comprehension abilities**. The mean reaction time is used as a summary score.
**The Trail Making Task – B (TMT-B)**	The task requires participants to draw lines between circles randomly distributed on a paper (whereby each circle contains a letter or a number). The participant is asked to connect the circles sequentially by alternating between numbers and letters.	This task **measures visual scanning, attentional set shifting and cognitive flexibility**. The time required to complete the task is measured and used as a summary score.
**Continuous Performance Test (CPT)**	Participants are presented with a series of continuously changing visual stimuli and the participant is required to respond by pressing a button when a stimulus is presented on the screen and to refrain from responding when a "non-target" stimulus (e.g., "X") is presented.	This task **measures sustained attention and response inhibition**. The total reaction time for correct responses is used a summary score.
**Wechsler Abbreviated Scale of Intelligence (WASI)**	Participants complete a series of tasks measuring visuo-spatial and problem-solving, abstract verbal and non-verbal reasoning, verbal expression, semantic knowledge, and verbal comprehension.	The scaled total IQ score is used as a summary **measure of general intelligence.**

Supplementary Table 2. Clinical, socio-demographic, and behavioural profiles of included VPT sample (n=116), relative to VPT excluded from whole sample (n=37).

Variable	VPT excluded (n=37)	VPT included (n=116)	p-value
**Age at assessment, years**	31.62 (3.39)	31.37 (3.71)	0.115
**Gestational age at birth, weeks**	28.00 (4.00)	30.00 (3.00)	0.024
**Birth weight, grams**	1183.00 (253.00)	1345.00 (512.00)	0.453
[a] **Perinatal brain injury (None: Minor: Major), n**	8:7:13	49:22:22	0.038
[b] **Participants' current socio-economic status (I – II: III: I-V: Student: Unemployed), n**	16: 7: 5: 1: 8	51: 41: 6: 1: 16	0.094
[b] **Parental socio-economic status at birth (I – II: III: I-V), n**	15:9:3	43:36:8	0.761
**Sex M:F, n**	24:13	66:50	0.506
[c] **COWAT, total words**	10.00 (7.25)	13.00 (5.75)	0.042
[c] **CANTAB – SOC, problems solved**	8.60 (3.00)	9.00 (2.75)	0.228
[c] **CANTAB – IED, total errors adjusted**	20.60 (41.75)	15.00 (25.50)	0.342
[c] **TMT-B, time to finish task**	100.50 (51.80)	73.50 (40.50)	0.007
[c] **CPT, total reaction time for correct responses**	430.90 (84.75)	417.50 (59.15)	0.226
[c] **WASI – full scale IQ**	100.70 (24.65)	106 .00(13.75)	0.097
[c] **CANTAB – MOT, reaction time**	717.50 (242.65)	691.00 (200.80)	0.990
[d] **PDI, total score**	24.50 (43.50)	21.50 (50.25)	0.680
[e] **AQ10, total score**	3.31 (3.00)	2.00 (2.44)	0.031
[f] **CAARMS, general psychopathology score**	2.50 (9.25)	2.00 (5.50)	0.133
[g] **GHQ, total score**	12.00 (6.00)	10.00 (6.00)	0.594
[c] **ERT, total correct**	53.50 (10.25)	56.60 (11.15)	0.092
[c] **SAS, total score**	1.49 (0.56)	1.58 (0.45)	0.867

Note. *Median (interquartile range) reported unless number of participants (n) is reported. [a] Perinatal brain injury rated from ultrasound scans: no haemorrhage (none), grade I – II periventricular haemorrhage without ventricular dilation (minor injury) and grade III – IV periventricular haemorrhage with ventricular dilation (major injury). [b] Socio-economic status occupation classifications. I: Higher managerial, administrative and professional occupations; II: Intermediate occupations, small employers and own account workers; III: Routine and manual occupations – lower supervisory and technical and semi-routine and routine occupations. Missing data for VPT excluded and included respectively: [c](n=9, n=22), [d](n=1, n=22), [e](n=10, n=19), [f](n=1, n=17), [g](n=0, n=9).*

Supplementary Table 3. VPT only – At-risk and Resilient behavioural subgroup clinical and socio-demographic profiles.

Variable	VPT Subgroup 1 – Resilient	VPT Subgroup 2 – At-risk	p-value	FDR p-value
**Age at assessment, years**	31.03 (3.34)	30.50 (3.60)	0.635	0.635
**Gestational age at birth, weeks**	30.00 (4.00)	30.00 (3.25)	0.187	0.262
**Birth weight, grams**	1382.50 (479.50)	1201.50 (498.75)	0.035	0.081
**[a] Perinatal brain injury, n (%)**			0.303	0.416
None	20 (48.78%)	26 (59.09%)		
Minor	12 (29.27%)	7 (15.91%)		
Major	8 (19.51%)	11 (25%)		
**[b] Participants' current socio-economic status, n (%)**				
I – II	28 (68.29%)	16 (36.36%%)	0.007	0.047
III	12 (29.27%)	17 (38.64%)		
IV – V	0 (0.00%)	2 (4.55%)		
Student	0 (0.00%)	1 (2.27%)		
Unemployed	1 (2.44%%)	8 (18.18%)		
**[b] Parental socio-economic status at birth, n (%)**			0.177	0.262
I – II	23 (56.10%)	16 (36.36%)		
III	12 (29.27%)	20 (45.46%)		
IV – V	4 (9.76%)	4 (9.09%)		
**Sex, n (%)**			0.029	0.081
Male	30 (73.17%)	21 (47.73%)		
Female	11 (26.83%)	23 (52.27%)		
**Total, n**	41	44		

Note. *Median (interquartile range) reported unless stated otherwise where number of participants (n) is reported alongside percentage (%). [a] Brain ultrasound scans were used to rate perinatal brain injury into three categories: no haemorrhage (no injury), grade I – II periventricular haemorrhage without ventricular dilation (minor injury) and grade III – IV periventricular haemorrhage with ventricular dilation (major injury). [b] Socio-economic status was categorised according to the Office of National Statistics, 1980 occupation classifications. I: Higher managerial, administrative and professional occupations; II: Intermediate occupations, small employers and own account workers; III: Routine and manual occupations – lower supervisory and technical and semi-routine and routine occupations.*

Supplementary Table 4. FT only – At-risk and Resilient behavioural subgroup socio-demographic profiles.

Variable	FT Subgroup 1 – Resilient	FT Subgroup 2 – At-risk	p-value	FDR p-value
**Age at assessment, years**	28.82 (3.36)	29.48 (5.31)	0.61	0.811
**[a] Participants' current socio-economic status, n (%)**			0.035	0.138
I – II	18 (60.00%)	14 (34.15%)		
III	9 (30.00%)	14 (34.15%)		
IV – V	0 (0.00%)	0 (0.00%)		
Student	1 (3.33%)	10 (24.39%)		
Unemployed	1 (3.33%)	3 (7.31%)		
**[a] Parental socio-economic status at birth, n (%)**			0.217	0.435
I – II	21 (70%)	17 (41.46%)		
III	4 (13.33%)	10 (24.39%)		
IV – V	1 (3.33%)	2 (4.88%)		
**Sex, n (%)**			0.831	0.831
Male	13 (43.33%)	20 (48.78%)		
Female	17 (56.67%)	21 (51.22%)		
**Total, n**	30	41		

Note. *Median (interquartile range) reported unless stated otherwise where number of participants (n) is reported alongside percentage (%).* [a] *Socio-economic status was categorised according to the Office of National Statistics, 1980 occupation classifications. I: Higher managerial, administrative and professional occupations; II: Intermediate occupations, small employers and own account workers; III: Routine and manual occupations – lower supervisory and technical and semi-routine and routine occupations.*

Supplementary Table 5. Two-way group interaction effects investigating whether VPT and FT group behavioural outcomes vary depending on data-driven behavioural subgrouping

	**Subgroup 1 – Resilient (n=71)**		**Subgroup 2 – At-risk (n=85)**		**Adj. p-value**	**Adj. FDR p-value**
	VPT (n=41)	*FT (n=30)*	*VPT (n=44)*	*FT (n=41)*		
**COWAT, total words**	14.00 (12.00)	14.00 (6.75)	12.00 (4.25)	14.00 (4.00)	0.178	0.578
**CANTAB – SOC, problems solved**	10.00 (9.00)	11.00 (1.00)	9.00 (2.00)	9.00 (2.00)	0.356	0.664
**CANTAB – IED, total errors adjusted**	14.00 (23.00)	9.00 (4.50)	23.00 (39.50)	13.00 (18.00)	0.829	0.829
**TMT-B, time to finish task**	62.00 (77.00)	57.50 (28.75)	77.00 (36.75)	84.00 (44.00)	0.676	0.732
**CPT, total reaction time for correct responses**	403.00 (432.00)	407.30 (47.65)	432.00 (65.00)	418.00 (57.00)	0.398	0.664
**WASI – full scale IQ**	107.00 (105.6)	117.00 (13.75)	105.6 (14.75)	109.00 (10.60)	0.011	0.148
**CANTAB – MOT, reaction time**	680.00 (705.5)	668.00 (137.75)	705.5 (262.75)	771.00 (182.00)	0.131	0.568
**PDI, total score**	14.00 (40.00)	9.50 (14.15)	40.00 (41.1)	41.80 (46.60)	0.408	0.664
**AQ10, total score**	1.99 (3.01)	2.00 (1.85)	3.01 (2.24)	3.00 (2.85)	0.568	0.711
**CAARMS, general psychopathology score**	1.00 (5.00)	0.00 (2.00)	5.00 (4.40)	4.00 (4.00)	0.368	0.664
**GHQ, total score**	8.00 (13.00)	7.90 (2.00)	13.00 (5.25)	13.00 (6.00)	0.601	0.711
**ERT, total correct**	54.00 (60.00)	61.50 (11.10)	60.00 (8.20)	62.00 (7.60)	0.043	0.282
**SAS, total score**	1.41 (1.81)	1.50 (0.34)	1.81 (0.46)	1.87 (0.50)	0.490	0.708

Note. *Median (interquartile range) reported. "Adj. p-value" corresponds to the p-value after adjusting for covariates (sex, age, socio-economic status) and "Adj. FDR p-value" corresponds to the Adj. p-value after correcting for multiple comparisons with FDR. Abbreviations: AQ10 = Autism Quotient; CANTAB = Cambridge Neurophysiological Test Automated Battery; CAARMS = Comprehensive Assessment of At-Risk Mental States; COWAT = Controlled Oral Word Association Test; CPT = Continuous Performance Test; ERT = Emotion Recognition Task; FT= full-term; GHQ = General Health Questionnaire; IED = Intra-Extra Dimensional Set Shift; MOT = Motor Screening Task; PDI = Peters Delusion Inventory; SAS = Social Adjustment Scale; SOC = Stockings of Cambridge; TMT-B = Trail Making Task B; VPT = very preterm; WASI = Wechsler Abbreviated Scale of Intelligence.*

Supplementary Table 6. Experimenting with p-value thresholds for NBS using two-tailed statistical testing.

Two-tailed effect of interest	p-value threshold	Number of identified components	Number of significant components	Significant component size	Significant component strength	FWE p-value
**VPT vs FT**	0.05	1	1	5244	2347.93	0.004
	0.01	1	1	1332	509.11	0.001
	0.001	22	1	153	50.08	<0.001
**At-risk vs Resilient**	0.05	1	0	n/a	n/a	n/a
	0.01	3	1	693	232.11	0.013
	0.001	29	1	27	12.27	0.013

Supplementary Table 7. VPT < FT – nodes with the highest number of connections within the significant NBS component.

Brain region	HCP-MMP atlas region	Number of edges	Percentage of edges
**Superior temporal gyrus (auditory association cortex)**	right Area STGa	36	2.453988
**Inferior parietal cortex**	left Area PGi	33	2.249489
**Inferior frontal cortex**	right Area 47l (47 lateral)	32	2.181322
**Superior parietal cortex**	left Medial IntraParietal Area	29	1.976823
**Orbitofrontal cortex**	right Area 47m	27	1.840491
**Inferior frontal cortex**	left Area IFJa	26	1.772324
**Orbitofrontal cortex**	left Area anterior 10p	26	1.772324
**Anterior cingulate and medial prefrontal cortex**	left Area p32	25	1.704158
**Inferior premotor**	left Rostral Area 6	25	1.704158
**Superior temporal gyrus (auditory association cortex)**	left Area STGa	25	1.704158
**Lateral occipital/posterior temporal visual area**	left Area PH	25	1.704158
**Superior parietal cortex**	left Area Lateral IntraParietal ventral	24	1.635992
**Orbitofrontal cortex**	left Area 47l (47 lateral)	24	1.635992
**Orbitofrontal cortex**	left Area 11l	24	1.635992
**Orbitofrontal cortex**	left Orbital Frontal Complex	24	1.635992
**Lateral temporal cortex**	left Area TE1 anterior	23	1.567825
**Dorsolateral prefrontal cortex**	left Superior Frontal Language Area	22	1.499659
**Inferior frontal cortex**	left Area IFSp	22	1.499659
**medial temporal lobe**	left ParaHippocampal Area 2	22	1.499659
**Medial temporal lobe**	right Entorhinal Cortex	22	1.499659
**Lateral temporal cortex**	right Area TG Ventral	21	1.431493
**Posterior cingulate cortex**	left Area 23d	20	1.363327
**Dorsolateral prefrontal cortex**	left Area 8B Lateral	19	1.29516
**Inferior parietal cortex**	left Area PGs	19	1.29516

**Dorsolateral prefrontal cortex**	right Area 8B Lateral	19	1.29516
**Orbitofrontal cortex**	right posterior OFC Complex	19	1.29516
**Dorsal stream visual cortex**	left Ventral Area 6	18	1.226994
**Anterior cingulate and medial prefrontal cortex**	right Area 9 Middle	18	1.226994
**Inferior parietal cortex**	right Area IntraParietal 2	18	1.226994
**Orbitofrontal cortex**	right Orbital Frontal Complex	17	1.158828
**Lateral occipital/posterior temporal visual area**	right Area PH	17	1.158828
**Anterior cingulate and medial prefrontal cortex**	right Area 25	17	1.158828
**Anterior cingulate and medial prefrontal cortex**	left Area 10r	16	1.090661
**Orbitofrontal cortex**	left Area anterior 47r	16	1.090661
**Orbitofrontal cortex**	left Area 13l	16	1.090661
**Orbitofrontal cortex**	right Area 11l	16	1.090661
**Superior temporal gyrus (auditory association cortex)**	right Area TA2	16	1.090661

Supplementary Table 8. VPT > FT – nodes with the highest number of connections within the significant NBS component.

Brain region	HCP-MMP atlas region	Number of edges	Percentage of edges
**Posterior opercular cortex**	left Frontal Opercular Area 4	30	3.118503
**Posterior opercular cortex**	right Frontal Opercular Area 4	30	3.118503
**Posterior cingulate cortex**	right Complex	26	2.702703
**Inferior parietal cortex**	left Area PF Complex	24	2.494802
**Inferior parietal cortex**	right Area IntraParietal 2	24	2.494802
**Orbitofrontal cortex**	right Area 10d	23	2.390852
**Inferior parietal cortex**	right Area PGs	22	2.286902
**Posterior opercular cortex**	right Area Frontal Opercular 5	22	2.286902
**Inferior parietal cortex**	right Area PGi	20	2.079002
**Anterior Cingulate and Medial Prefrontal Cortex**	left Area 10r	18	1.871102
**Posterior cingulate cortex**	right Parieto-Occipital Sulcus Area 1	18	1.871102
**Dorsolateral prefrontal cortex**	right Area posterior 9-46v	18	1.871102
**Superior temporal gyrus (auditory association cortex)**	right Area STGa	18	1.871102
**Lateral temporal cortex**	right Area TE1 Middle	18	1.871102
**Superior temporal gyrus (auditory association cortex)**	left Area STGa	16	1.663202
**Inferior parietal cortex**	right Area PFm Complex	16	1.663202
**Posterior opercular cortex**	left Area Frontal Opercular 5	15	1.559252
**Posterior cingulate cortex**	right Area 31pd	15	1.559252
**Anterior Cingulate and Medial Prefrontal Cortex**	right Anterior 24 prime	14	1.455301
**Anterior Cingulate and Medial Prefrontal Cortex**	right Area a24	14	1.455301
**Posterior cingulate cortex**	left Area dorsal 23 a+b	13	1.351351
**Posterior cingulate cortex**	left Area 31p ventral	13	1.351351
**Superior parietal cortex (medial)**	left Medial Area 7A	13	1.351351
**Anterior Cingulate and Medial Prefrontal Cortex**	left Area Posterior 24 prime	13	1.351351

**Anterior Cingulate and Medial Prefrontal Cortex**	right Area 10r	13	1.351351
**Dorsolatetal prefrontal cortex**	right Area 8C	13	1.351351
**Posterior opercular cortex**	right Area 43	13	1.351351
**Posterior cingulate cortex**	left Retrosplenial Complex	12	1.247401
**Inferior parietal cortex**	left Area PGi	12	1.247401
**Superior parietal cortex (medial)**	right Medial Area 7A	12	1.247401
**Anterior Cingulate and Medial Prefrontal Cortex**	right Area 9 Middle	12	1.247401
**Temporo-parietal-occipital junction**	right TemporoParietoOccipital Junction 2	12	1.247401
**Orbitofrontal cortex**	right Area posterior 10p	12	1.247401

Supplementary Table 9. At-risk < Resilient – nodes with the highest number of connections within the significant NBS component.

Brain region	HCP-MMP atlas region	Number of edges	Percentage of edges
**Insular cortex**	right Posterior Insular Area 2	32	3.846154
**Frontal opercular cortex**	left Area OP4/PV	25	3.004808
**Frontal opercular cortex**	left Frontal Opercular Area 4	24	2.884615
**Insular cortex**	right Middle Insular Area	24	2.884615
**Frontal opercular cortex**	left Frontal Opercular Area 2	23	2.764423
**Posterior opercular cortex**	right Area OP4/PV	23	2.764423
**Inferior frontal cortex**	left Area 44	22	2.644231
**Lateral occipital/posterior temporal visual area**	right Area PH	21	2.524038
**Insular cortex**	left Posterior Insular Area 2	20	2.403846
**Lateral temporal cortex**	left Area TE2 anterior	19	2.283654
**Superior premotor cortex**	right Dorsal area 6	18	2.163462
**Lateral temporal cortex**	left Area TG dorsal	15	1.802885
**Posterior opercular cortex**	right Area 43	15	1.802885
**Temporo-parieto-occipital junction**	left Superior Temporal Visual Area	14	1.682692
**Supplementary motor area**	left Supplementary and Cingulate Eye Field	14	1.682692
**Insular cortex**	left Insular Granular Complex	14	1.682692
**Lateral temporal cortex**	right Area TF	14	1.682692
**Posterior opercular cortex**	left Area OP2-3/VS	13	1.5625
**Anterior Cingulate and Medial Prefrontal Cortex**	left Area 25	13	1.5625
**Primary somatosensory cortex**	right Primary Sensory Cortex	13	1.5625
**Posterior opercular cortex**	right Area OP1/SII	12	1.442308
**Insular cortex**	right Area 52	12	1.442308
**Primary somatosensory cortex**	left Primary Sensory Cortex	11	1.322115
**Insular cortex**	left Middle Insular Area	11	1.322115

**Frontal opercular cortex**	left Frontal Opercular Area 3	11	1.322115
**Superior temporal sulcus (auditory association cortex)**	left Area STSd anterior	11	1.322115
**Lateral temporal cortex**	left Area TF	11	1.322115
**Anterior Cingulate and Medial Prefrontal Cortex**	right Area 8BM	11	1.322115
**Posterior opercular cortex**	right Area OP2-3/VS	11	1.322115
**Frontal opercular cortex**	right Frontal Opercular Area 3	11	1.322115
**Posterior opercular cortex**	left Area PFcm	10	1.201923
**Posterior opercular cortex**	left Frontal Opercular Area 1	10	1.201923
**Frontal opercular cortex**	right Frontal Opercular Area 2	10	1.201923
**Superior temporal sulcus (auditory association cortex)**	right Area STSd anterior	10	1.201923
**Lateral temporal cortex**	right Area TE1 anterior	10	1.201923

Supplementary Table 10. Sensitivity analyses – NBS component results at p-NBS-threshold = 0.001.

	Edges, n (% of all possible connections)	Nodes, n (% of all regions)	Component strength, T-stat	FWE p-value
VPT < FT	221 (0.32%)	179 (47.86%)	77.89	0.001
VPT > FT	42 (0.060%)	29 (7.75%)	16.36	0.001
At-risk < Resilient	60 (0.086%)	52 (13.90%)	24.84	0.006

References

1. Tustison NJ, Avants BB, Cook PA, et al. N4ITK: Improved N3 Bias Correction. *IEEE Trans Med Imaging*. 2010;29(6):1310-1320. doi:10.1109/TMI.2010.2046908

2. Avants BB, Epstein CL, Grossman M, Gee JC. Symmetric diffeomorphic image registration with cross-correlation: evaluating automated labeling of elderly and neurodegenerative brain. *Med Image Anal*. 2008;12(1):26-41. doi:10.1016/j.media.2007.06.004

3. Zhang Y, Brady M, Smith S. Segmentation of brain MR images through a hidden Markov random field model and the expectation-maximization algorithm. *IEEE Trans Med Imaging*. 2001;20(1):45-57. doi:10.1109/42.906424

4. Dale AM, Fischl B, Sereno MI. Cortical Surface-Based Analysis: I. Segmentation and Surface Reconstruction. *NeuroImage*. 1999;9(2):179-194. doi:10.1006/nimg.1998.0395

5. Klein A, Ghosh SS, Bao FS, et al. Mindboggling morphometry of human brains. *PLOS Comput Biol*. 2017;13(2):e1005350. doi:10.1371/journal.pcbi.1005350

6. Fonov V, Evans AC, Botteron K, Almli CR, McKinstry RC, Collins DL. Unbiased Average Age-Appropriate Atlases for Pediatric Studies. *NeuroImage*. 2011;54(1):313-327. doi:10.1016/j.neuroimage.2010.07.033

7. Jenkinson M, Bannister P, Brady M, Smith S. Improved optimization for the robust and accurate linear registration and motion correction of brain images. *NeuroImage*. 2002;17(2):825-841. doi:10.1016/s1053-8119(02)91132-8

8. Greve DN, Fischl B. Accurate and robust brain image alignment using boundary-based registration. *NeuroImage*. 2009;48(1):63-72. doi:10.1016/j.neuroimage.2009.06.060

9. Power JD, Mitra A, Laumann TO, Snyder AZ, Schlaggar BL, Petersen SE. Methods to detect, characterize, and remove motion artifact in resting state fMRI. *NeuroImage*. 2014;84:320-341. doi:10.1016/j.neuroimage.2013.08.048

10. Behzadi Y, Restom K, Liau J, Liu TT. A component based noise correction method (CompCor) for BOLD and perfusion based fMRI. *NeuroImage*. 2007;37(1):90-101. doi:10.1016/j.neuroimage.2007.04.042

11. Satterthwaite TD, Elliott MA, Gerraty RT, et al. An improved framework for confound regression and filtering for control of motion artifact in the preprocessing of resting-state functional connectivity data. *NeuroImage*. 2013;64:240-256. doi:10.1016/j.neuroimage.2012.08.052

12. Lanczos C. Evaluation of Noisy Data. *J Soc Ind Appl Math Ser B Numer Anal*. 1964;1(1):76-85. doi:10.1137/0701007

13. Abraham A, Pedregosa F, Eickenberg M, et al. Machine learning for neuroimaging with scikit-learn. *Front Neuroinformatics*. 2014;8. Accessed April 21, 2023. https://www.frontiersin.org/articles/10.3389/fninf.2014.00014
